# Genome sequence of the Chinese white wax scale insect *Ericerus pela*: the first draft genome for the Coccidae family of scale insects

**DOI:** 10.1093/gigascience/giz113

**Published:** 2019-09-13

**Authors:** Pu Yang, Shuhui Yu, Junjun Hao, Wei Liu, Zunling Zhao, Zengrong Zhu, Tao Sun, Xueqing Wang, Qisheng Song

**Affiliations:** 1 Research Institute of Resource Insects, Chinese Academy of Forestry, Key Laboratory of Cultivating and Utilization of Resource Insects of State Forestry Administration, Kunming 650224, China; 2 College of Agriculture and Life Sciences, Kunming University, Kunming 650214, China; 3 State Key Laboratory of Genetic Resources and Evolution, Laboratory of Evolutionary and Functional Genomics, Kunming Institute of Zoology, Chinese Academy of Sciences, Kunming 650223, Yunnan, China; 4 State Key Laboratory of Rice Biology/Key Laboratory of Molecular Biology of Crop Pathogens and Insects, Ministry of Agriculture/Institute of Insect Sciences, Zhejiang University, Hangzhou 310058, China; 5 Division of Plant Sciences, University of Missouri, Columbia, MO 65211, USA

**Keywords:** *Ericerus pela*, Chinese white wax scale insect, wax secretion, adaptation, genome

## Abstract

**Background:**

The Chinese white wax scale insect, *Ericerus pela*, is best known for producing wax, which has been widely used in candle production, casting, Chinese medicine, and wax printing products for thousands of years. The secretion of wax, and other unusual features of scale insects, is thought to be an adaptation to their change from an ancestral ground-dwelling lifestyle to a sedentary lifestyle on the higher parts of plants. As well as helping to improve its economic value, studies of *E. pela* might also help to explain the adaptation of scale insects. However, no genomic data are currently available for *E. pela*.

**Findings:**

To assemble the *E. pela* genome, 303.92 Gb of data were generated using Illumina and Pacific Biosciences sequencing, producing 277.22 Gb of clean data for assembly. The assembled genome size was 0.66 Gb, with 1,979 scaffolds and a scaffold N50 of 735 kb. The guanine + cytosine content was 33.80%. A total of 12,022 protein-coding genes were predicted, with a mean coding sequence length of 1,370 bp. Twenty-six fatty acyl-CoA reductase genes and 35 acyltransferase genes were identified. Evolutionary analysis revealed that *E. pela* and aphids formed a sister group and split ∼241.1 million years ago. There were 214 expanded gene families and 2,219 contracted gene families in *E. pela*.

**Conclusion:**

We present the first genome sequence from the Coccidae family. These results will help to increase our understanding of the evolution of unique features in scale insects, and provide important genetic information for further research.

## Data Description

**Figure 1:**
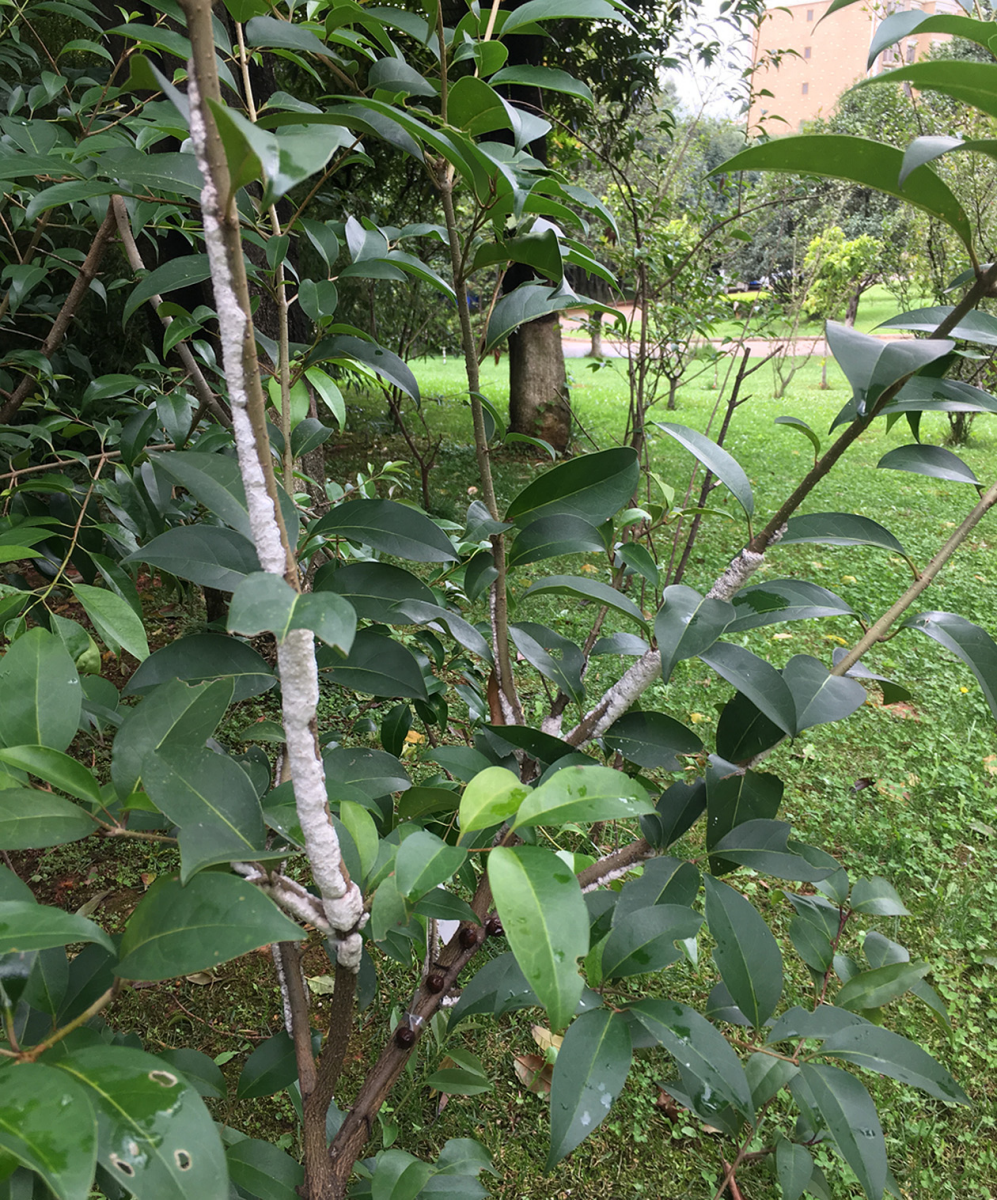
The branches of a Chinese glossy privet tree covered by a white wax layer secreted by *Ericerus pela. E. pela* insects gather and assemble one by one on the branches, and secrete wax continuously to form a layer that covers their bodies. The *E. pela* individuals are not visible because they are covered by the wax layer.

The Chinese white wax scale insect (*Ericerus pela*), silkworm (*Bombyx mori*), and honeybee (*Apis cerana*) are 3 traditionally domesticated insect species in China. *E. pela* (NCBI:txid931557) is best known for its wax production (Fig. 1). The useful properties of the wax secreted by this insect mean that it is harvested for candles and polishes, as well as for food, medicine, and the cosmetics industries in China and Japan [1–7]. Insect wax–based materials are derived from white wax (produced by *E. pela*) and yellow wax (produced by *A. cerana*). However, *E. pela* is the main wax producer. Each individual white scale insect produces, on average, ∼0.45 mg of wax on the host tree, glossy privet (*Ligustrum lucidum*) [8]. Annual wax production ranges from 300 to 500 tons and creates revenue of ∼60–100 million Chinese yuan. The long-chain alcohols made from white wax and other white wax products have additional economic value.

*E. pela* is a typical scale insect, of which wax secretion is the most striking feature. There are 2 major groups, archaeococcoids and neococcoids, and ∼8,000 species of scale insect. The neococcoids, which is the most recently evolved group, includes 17 families, including the Coccidae, Pseudococcidae, and Dactylopiidae. Some of these species are important pests or resource insects [9–11]. *E. pela* belongs to the family Coccidae and is the only species in the genus *Ericerus*. The “scale” part of the common name derives from the protective cover commonly formed by wax secretions on this type of insect. The wax secretions have antimicrobial activity and hydrophobic properties, which serve protective functions. The secretions of some scale insect species have been applied in industrial fields [2, 12].

In the husbandry process, insects are fed and deposit their secretions on the branches of certain species of *Ligustrum* (privet) trees. These secretions are harvested and boiled in water to extract the raw wax. At the end of the process, the leftover insect bodies are used as animal feed.

Before the diversification of angiosperms, ancestral scale insects were initially ground leaf-litter dwellers. Many of the special features of scale insects are legacy adaptations to this ancestral lifestyle. With the increasing predominance of seed plants, scale insects evolved to inhabit the aerial parts of seed plants. This exposed living environment, with its associated increased risk of predation, placed selective pressure on scale insects. Their protective cover enhances their survival. Wax secretion is a special survival strategy that arose from adaptation to a sedentary lifestyle on host plants [12, 13].

Apart from wax secretion, perhaps the best-known feature of scale insects is their sexual dimorphism. The females have reduced or lost appendages, and their bodies are spherical. Males and females are sexually dimorphic in many aspects and appear to be 2 different species [1, 4, 5]. Sexual dimorphism is beneficial for male courtship and female reproduction in *E. pela*, and makes full use of the resources within a habitat. Because it is a typical scale insect, the study of *E. pela* provides opportunities for investigating the mechanism of wax secretion, as well as the adaptive evolution of scale insects in specific environments.

Using transcriptome and gene expression profiles, and gene cloning and expression techniques, we previously studied the molecular biology of white wax biosynthesis in these scale insects, as well as their sexual dimorphism, antifreeze biology, and microbial symbiosis [1–7, 12]. However, no genomic data are currently available for *E. pela*, which hinders further study of the biology and genetics of this insect. In this study, we constructed 7 libraries, with different insert sizes for Illumina and Pacific Biosciences (PacBio) sequencing, and assembled the *E. pela* genome. This information will aid breeding and variety selection of this species and will be useful in species conservation. In addition, the genome will provide insight into the phylogenetic relationships between *E. pela* and other insects in the tree of life, and the relationships between families of other scale insects. The data will inform insect systematics and evolutionary research and could help to fill gaps in phylogenetic research and the genomic basis of insect diversity.

### Sample preparation and library construction

Individual *E. pela* insects from the Kunming geographical population were reared at the Research Institute of Resource Insects, Kunming, China. Each individual can produce thousands of offspring. The offspring produced by 1 individual were reared on 1 host tree (*L. lucidum*) planted in 1 flowerpot. To remove microbial symbionts, female adults were washed 3 times with double-distilled water for 5 min and then dissected in phosphate-buffered saline (pH 7.4) under a stereomicroscope. The cuticle, gut, ovaries, etc., were then detached carefully, and the remaining tissue was washed 3 times in cold phosphate-buffered saline for 5 min. More than 20 individuals were used for genomic DNA isolation.

The samples were crushed to powder in a mortar with liquid nitrogen. Then, 3 mL of lysis buffer (10 mM Tris-HCl, 400 mM NaCl, 2 mM ethylenediaminetetraacetic acid [EDTA]-2Na, and 0.8 M guanidine hydrochloride), 20 µL of proteinase K (50 mg/mL), and 200 µL of sodium dodecyl sulfate were added. The solution was incubated at 56°C for 45 min. Next, 3.5 mL of isolation buffer (240 mL chloroform, 10 mL isoamyl alcohol, and 250 mL Tris-phenol) was added to the solution before centrifugation at 4,700 rpm for 10 min. The supernatant was transferred to a new tube, and the isolation step was repeated. Then, 3 mL of isopropyl alcohol (precooled to –20°C) was added to the supernatant. The DNA precipitate was obtained and washed with 70% (v/v) ethanol. Then, 100 μL of Tris-EDTA was added to dissolve the DNA after the ethanol had completely volatilized. To degrade the RNA, 2 μL of RNase A (10 mg/mL) was added. The DNA concentration was determined with a NanoDrop 8000 spectrophotometer (Thermo Fisher Scientific, Waltham, MA, USA) and a Qubit fluorometer (Invitrogen, Carlsbad, CA, USA). DNA quality was tested by pulsed-field gel electrophoresis.

Six libraries with a gradient of insert sizes (200, 350, and 500 bp and 2, 5, and 10 kb) (Additional Table S1) were constructed for second-generation sequencing. For each of the 3 small-insert-size libraries, 2 μg of genomic DNA (concentration ≥20 ng/µL) was separately broken into 200-bp, 350-bp, or 500-bp fragments by an ultrasonic processor. After end-repair, A-tail addition, sequence adaptor addition, purification, and PCR, the libraries were constructed according to the manufacturer's protocol (Illumina, San Diego, CA, USA).

To construct each of the 2-kb, 5-kb, and 10-kb libraries, ∼20 µg of genomic DNA was fragmented by an ultrasonic processor. After end-repair, the fragments were biotinylated. Target fragments were selected on an agarose gel. To capture circular self-ligated DNA fragments, the DNA was fragmented again and biotinylated. After purification with M-280 streptavidin Dynabeads (Invitrogen), the fragments were end-repaired, the A-tail was added, and the adaptor was ligated to the fragments. PCR amplification was performed, and 400–600-bp products were selected on an agarose gel and purified. The library was then quantitated with a Qubit 2.0 fluorometer and diluted to 1.5 ng/µL. The insert size of the libraries was detected on an Agilent 2100 Bioanalyzer (Agilent, Santa Clara, CA, USA). Real-time quantitative PCR was performed to quantify the libraries. Libraries were then sequenced on Illumina HiSeq 2500 (the 3 small-insert-size libraries), HiSeq 2000 (2-kb, 5-kb libraries), and HiSeq x-ten (10-kb library) system (Illumina).

A PacBio 20K library was constructed for third-generation sequencing. Approximately 10 µg of genomic DNA was broken into fragments of ∼17 kb. The fragments were digested by exonuclease VII, damage-repaired, and end-repaired. The fragments were then ligated with adaptors overnight. After enzyme digestion and fragment size selection, the library was constructed. The templates were annealed with primers, subjected to polymerase binding, and sequenced on a PacBio Sequel system (Menlo Park, CA, USA) using the MagBead loading model.

### Data processing and genome evaluation and assembly

Sequence quality was assessed by sequence quality distribution, error rate distribution, and guanine-cytosine content analyses. Raw data were filtered as follows: (i) adaptor sequences were removed; (ii) when the N content in the reads obtained from single-end sequencing was >10%, the paired reads were removed; and (3) when the percentage of low-quality bases in the reads obtained from single-end sequencing was >50%, the paired reads were removed.

Error correction was performed to correct the filtered data of the 3 small-insert-size libraries. Sequencing errors can result in new *k*-mers with low frequencies. A *k*-mer frequency of 10 was considered the cut-off between low and high frequencies for error correction. Some bases in the reads that had a low frequency were corrected to ensure that the reads had a high frequency [14].

**Figure 2:**
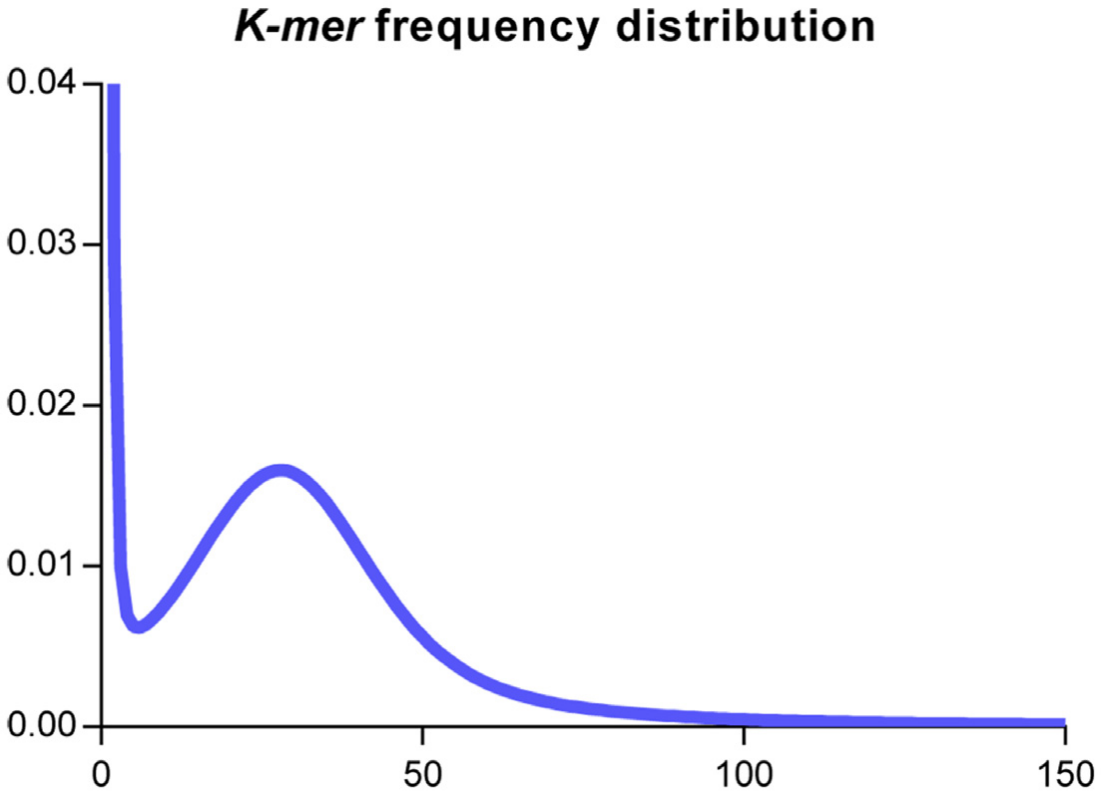
Read distribution obtained from 17-mer analysis. X-axis shows the sequencing depth. Y-axis shows the proportion of *k*-mers from a sequencing depth to the total number of *k*-mers.

The *k*-mer method [14] was used to examine *E. pela* genome size and heterozygosity before genome assembly. To generate a 17-mer depth frequency curve, 25,370,340,375 bp of high-quality data were used (Fig. 2). There was 1 peak in the curve, located at ∼28 bp. The total *k*-mer number was 22,122,936,807. The genome size was calculated to be 0.79 Gb, according to the following formula:

$$\begin{equation*}
{\rm{Genome\,Size = k-mer\_num/Peak\_depth}}
\end{equation*}
$$

(Additional Table S2) [14]. There was no heterozygosity peak in the *E. pela* genome (Fig. 2).

The filtered data were first assembled into contigs using Platanus software (1.2.1, Platanus, RRID:SCR_015531) [15]. Then, contigs and PacBio sequence data were used to assemble scaffolds using the DBG2OLC method [16]. A total of 247 Gb of second-generation data and 30 Gb of third-generation data were used for assembly. Owing to the high error rate of PacBio sequencing, the scaffolds had many minor errors. Error correction was performed using the second-generation and third-generation sequencing data. Preliminary corrections were conducted by Pilon 1.22 software (Pilon, RRID:SCR_014731), based on the alignment of second-generation sequencing data with the assembled sequences. The scaffold was further constructed using SSPACE software (SSPACE, RRID:SCR_005056) [17], and PacBio sequence data were used to fill the gaps in the scaffold using the PBJelly program (PBJelly, RRID:SCR_012091) [18]. Finally, Polish software was used to perform the second error correction.

The *E. pela* genome was finally assembled into a 0.66-Gb genome, consisting of 1,979 scaffolds. The N50 of the scaffolds was 735,622 bp, and the N50 of the contigs was 660,240 bp (Table 1). The genome size was similar to that of *Bemisia tabaci* (658 Mb) [19] and *Sogatella furcifera* (720 Mb) [20], smaller than that of *Nilaparvata lugens* (1,141 Mb) [21], and larger than that of *Acyrthosiphon pisum* (464 Mb) [22].

**Table 1:** Data about the genome assembly of *Ericerus pela*

Type	Contig		Scaffold	
	Size (bp)	No.	Size (bp)	No.
N90	146,803	1,066	160,747	964
N80	279,168	744	309,099	673
N70	420,664	554	455,420	502
N60	530,834	414	594,019	375
N50	660,240	302	735,622	275
Maximum length	4,102,106		4,102,106	
Total length	660,732,850		660,870,788	
Total No. (≥100 bp)		2,173		1,979
Total No. (≥2 kb)		2,168		1979

The genome assembly statistical analysis included contig and scaffold.

The guanine + cytosine content of the *E. pela* genome was 33.80%, which was similar to that of *N. lugens* (34.60%) and *S. furcifera* (31.60%). However, it was lower than that of *B. tabaci* (39.00%) and higher than that of *A. pisum* (29.60%).

### Genome assembly analysis

After genome assembly, the sequencing depth was calculated by SOAP coverage 2.27 (SOAP, RRID:SCR_000689) [23]. Four transcriptome data sequences [3–6] were used as query sequences and mapped to the assembled genome sequence. Coverage of the assembled sequences by transcriptome sequences was tested. BUSCO software (version 3, BUSCO, RRID:SCR_015008) [24] was used to evaluate coding gene completeness.

Reads from 4 transcriptomes were mapped to the *E. pela* genome. The results showed that 88.41%, 86.67%, 86.26%, and 91.03% of the reads from the 4 transcriptomes [3–6] were mapped to the genome.

### Repeat sequence annotation

Tandem repeat sequences were identified using TRF software [25]. Interspersed repeat sequences (transposons) were identified using RepeatMasker and RepeatProteinMask software, based on the Repbase database. *De novo* prediction was performed using RepeatMasker software, which is based on the database from RepeatModeler (RepeatModeler, RRID:SCR_015027). Non-redundant results were obtained after all of the results predicted above were combined, and overlapping results were removed.

The *E. pela* genome contained 55.06% repeat sequences (Table 2), which is more than in *N. lugens* (48.6%) [21] and *A. pisum* (33.3%) [22].

**Table 2:** Transposable element (TE) content in the *Ericerus pela* genome

Type	Repbase TEs		TE proteins		*de novo*		Combined TEs	
	Length (kb)	% in genome	Length (kb)	% in genome	Length (kb)	% in genome	Length (kb)	% in genome
DNA	6,232	0.9430	1,231	0.1863	34,058	5.1535	38,500	5.8256
LINE	1,633	0.2470	99	0.0149	2,996	0.4533	4,607	0.6971
SINE	20	0.0030	0	0	1,477	0.2235	1,492	0.2257
LTR	26,102	3.9497	53,919	8.1588	130,662	19.7712	141,133	21.3555
Other	16	0.0024	0	0	0	0	16	0.0024
Unknown	0	0	0	0	197,674	29.9112	197,674	29.9112
Total	32,011	4.8437	55,246	8.3596	359,819	54.4462	363,875	55.0599

Abbreviations: LINE, long interspersed nuclear element; LTR, long terminal repeat; SINE, short interspersed nuclear element; TE, transposable element.

The transposable elements (TEs) in *E. pela*, identified through *de novo* prediction, showed a peak sequence shift compared with those identified through a homology-based approach (Additional Figure S1). This suggests the recent evolution of DNA transposons, which is similar to the pattern observed for the genome of *N. lugens* [21].

### Gene prediction and annotation

Protein homology-based gene prediction was performed using BLASTN (BLASTN, RRID:SCR_001598) [26]. The genomes of 8 insect species (*A. pisum, Apis mellifera, B. tabaci, B. mori, Drosophila melanogaster, Nasonia vitripennis, Pediculus humanus*, and *Tribolium castaneum*) were selected as references for homology prediction. Alignment was performed using BLAST, with a TBLASTN e-value cut-off of 1e–05. Alignment results were ordered and filtered using Solar software (SOLAR, RRID:SCR_000850), according to an alignment rate of 0.33. Then, GeneWise (GeneWise, RRID:SCR_015054) alignment was performed [27]. Augustus software (Augustus, RRID:SCR_008417) [28] was used for *de novo* prediction. Complete gene sets were selected separately from the aforementioned homology prediction results. Then, 2,000 gene sets were selected at random from the 8 species, and perfect genes were selected for Augustus training. The Augustus prediction result for the *E. pela* genome was 19,941 genes. In addition, the transcripts were used to supplement the gene sets. RNA-sequencing data were aligned with the genome. StringTie software (StringTie, RRID:SCR_016323) was used for assembly, Cuffmerge (Cuffmerge, RRID:SCR_015688) was used to merge the results and delete redundant reads, and Cuffcompare was used to compare results and obtain the transcript set [29]. Genes predicted by these 3 methods were then integrated into 1 non-redundant gene set, and 1 more complete gene set using GLEAN software [30]. Finally, protein databases (SwissProt, TrEMBL, KEGG, InterPro, and Gene Ontology [GO]) were used to annotate the protein functions of the gene sets. Our genome was compared aginst the database of Arthropoda genomes. The complete BUSCO score was 83.2%.

A total of 12,022 protein-coding genes were predicted using a combination of *de novo*, RNA-sequencing, and homolog prediction. The number of predicted genes in *E. pela* was similar to that in *D. melanogaster* (13,689), *P. humanus* (10,769), and *A. mellifera* (10,660), and lower than that in *N. lugens* (27,571) [21], *A. pisum* (33,267) [22], *S. furcifera* (21,254) [20], and *B. tabaci* (20,786) [19]. A total of 87.99% of the gene sets genes were functionally annotated (Table 3).

**Table 3:** Functional annotations of the *Ericerus pela* genome

Annotations	No. (%)
Total	12,022 (100)
Nr-Annotated	9,176 (76.33)
Nt-Annotated	10,255 (85.30)
Swissprot-Annotated	8,536 (71.00)
KEGG-Annotated	8,628 (71.77)
COG-Annotated	4,398 (36.58)
TrEMBL-Annotated	10,320 (85.84)
Interpro-Annotated	9,609 (79.93)
GO-Annotated	3,875 (32.23)
Overall	10,578 (87.99)

Abbreviations: COG, cluster of ortholog genes; GO, gene ontology Nr, NCBI non-redundant protein sequences; Nt, NCBI non-redundant nucleotide sequences.

The mean coding sequence length in *E. pela* was 1,370 bp, which was slightly longer than that in *N. lugens* (1,135 bp) [21] and shorter than that in *S. furcifera* (1,577 bp) [20]. The mean intron length was 1,673 bp.

Fatty acyl-CoA reductase genes (*far*) and acyltransferase genes are related to wax secretion [3]. Twenty-six *far* genes were found in the *E. pela* genome, which is a moderate number compared with that in other Hemiptera insects (*A. pisum*: 34; *Diuraphis noxia*: 27; *Diaphorina citri*: 46; and *B. tabaci*: 25). However, a total of 35 acyltransferase gene family members were identified in the *E. pela* genome, which was larger than the number identified in the other 4 hemipteran insects (*A. pisum*: 15; *D. noxia*: 13; *D. citri*: 16; and *B. tabaci*: 25).

### Noncoding RNA annotation

Transfer RNA (tRNA) was identified according to structure, using tRNAscan-SE software (tRNAscan-SE, RRID:SCR_010835) [31]. Ribosomal RNA (rRNA) was identified by BLASTN alignment, using the rRNA sequences from closely related species as query sequences. MicroRNA and small nuclear RNA were predicted using INFERNAL software (Infernal, RRID:SCR_011809) in Rfam, according to the covariance model in Rfam [32]. Noncoding RNA, including rRNA, tRNA, nuclear RNA, and microRNA, was identified in the *E. pela* genome (Additional Table S3).

### Gene phylogenomics

The gene sets of 14 species were filtered to obtain high-quality gene sets. Gene clusters were identified using OrthoMCL software [33]. Single-copy and multiple-copy gene families were obtained by homolog identification and gene family cluster analysis. Sixty-five single-copy gene families were identified across the 14 species.

Single-copy gene families were arrayed as a supergene after multiple sequence alignment. This supergene was used to construct a phylogenetic tree [34, 35]. Species divergence times were calculated according to the molecular clock, based on the 4-fold-degenerate codons of the single-copy gene families [36–42]. The phylogenetic tree constructed on the basis of single-copy orthologs showed that *E. pela* formed a sister group with aphids (*A. pisum* and *D. noxia*), and this group formed a sister group with a psyllid (*D. citri*) (Additional Figure S2). This indicates that scale insects and aphids evolved more recently than other hemipteran insects, such as white flies and plant lice.

**Figure 3:**
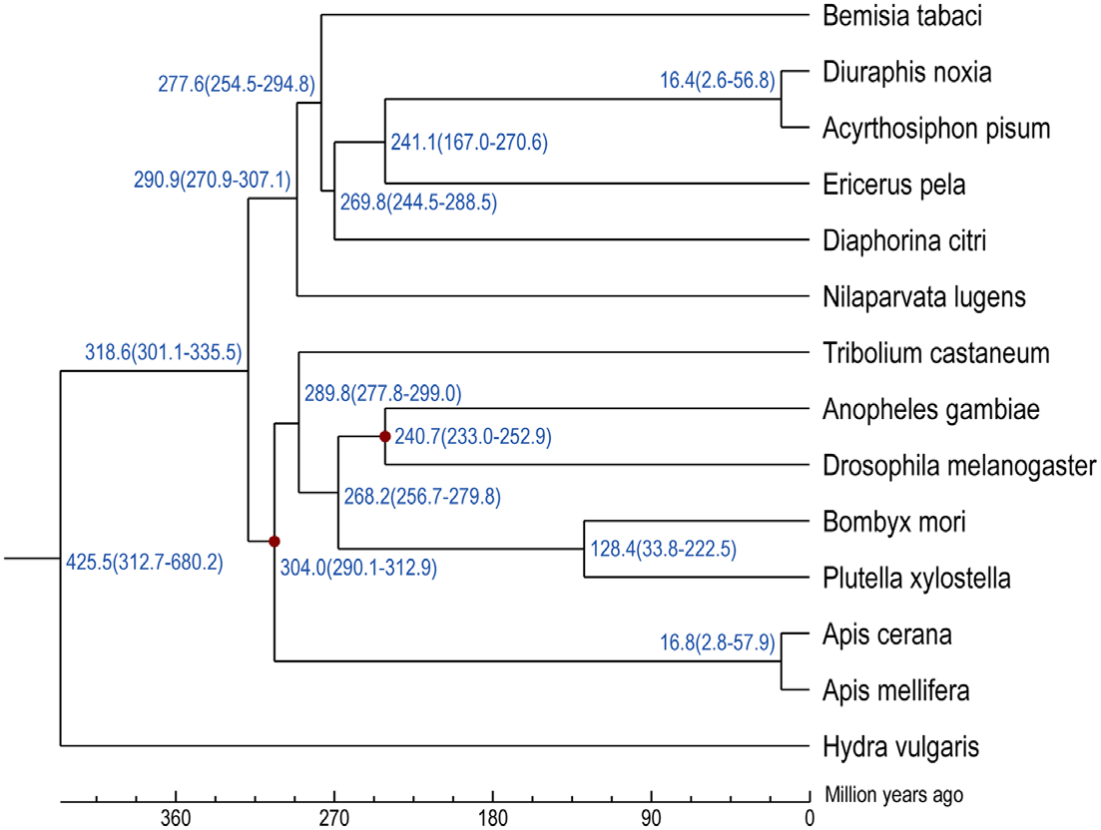
The estimated divergence time between 14 arthropod species. The number at each node is the divergence time in million years ago (MYA). The 2 divergence times used for calibration of branch divergence times are marked with red dots at the node.

It was estimated that *E. pela* and aphids diverged ∼241.1 million years ago (MYA) (Fig. 3). Fossils have shown that the ancestors of scale insects exhibited modern morphology by the Lower Cretaceous period (65–137 MYA). It is likely that the evolution of scale insects occurred even earlier—possibly during the mid-Mesozoic period (60–250 MYA) or before. Ancestral scale insects originally lived in the leaf-litter layer and sucked plant roots, similar to modern thrips [13]. Angiosperms diversified until 90–130 MYA, so ancient scale insects must have fed on gymnosperms or fungi. Many of the unique features of scale insects, such as wax secretion and appendage reduction, are considered legacies from their ground-dwelling ancestors. The phylogenetic tree indicated that *E. pela* diverged from aphids ∼241.1 MYA. This timescale supports the early evolution of scale insects. Furthermore, it is consistent with the idea that the specializations of ancestral scale insects led to myriad unusual features and that a subsequent parasitic lifestyle on angiosperms further favored appendage reduction and wax secretion.

**Figure 4:**
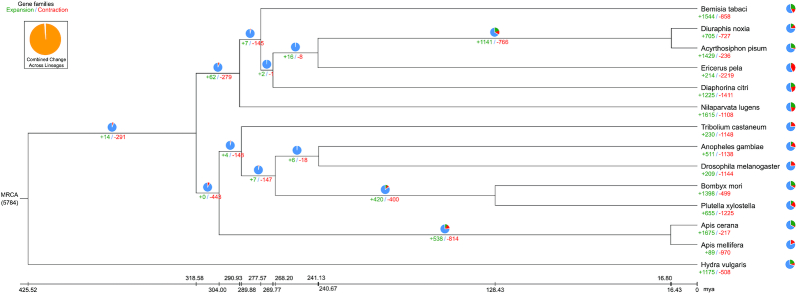
Phylogenetic tree showing gene family contraction and expansion in *Ericerus pela* compared with 13 other species. The green numbers under the branch represent the number of expanded gene families, and the red numbers represent the number of contracted gene families. The green part of the pie is the percentage of expanded gene families, the red part is the percentage of contracted gene families, and the blue part is the percentage of gene families that remain unchanged.

TreeFam [43] was used to define gene families comprising a group of genes descended from 1 gene of the most recent common ancestor [44]. CAFÉ software was used to detect gene family expansion and contraction (*P* < 0.05) [45]. There were 214 expanded gene families and 2,219 contracted gene families in *E. pela* (Fig. 4). Two gene families were completely absent from the *E. pela* genome (Additional Table S4): these families were related to RNA-directed DNA polymerase from mobile element jockey-like, and glutathione S-transferases (GSTs). GSTs are thought to be important in stress response and insecticide/drug resistance [46–48]. The gene family contraction of GSTs in *E. pela* may be explained by the relaxation of natural selection because of the protective function of the white wax layer.

In addition, we found 1 gene family with members almost unique to *E. pela* (Additional Table S5), related to aldo-keto reductases (AKRs). AKRs reduce a variety of carbonyl-containing compounds to corresponding alcohols in the presence of NADPH [49–51]. Alcohols are 1 of the 2 substrates used to form wax esters.

Expanded and contracted gene families were selected for further functional analysis. The Blast2GO (Blast2GO, RRID:SCR_005828) and BLAST programs were used to perform GO [52] and KEGG orthology analyses [53]. GO analysis showed that the contracted genes were mainly involved in functions such as microtubule-based movement, and movement of cell or subcellular components, which may be responsible for the insect's sedentary lifestyle on plants. Expanded genes were mainly related to nucleic acid binding, organic cyclic compound binding, and protein dimerization activity. KEGG analysis indicated that some contracted genes were related to cardiac muscle contraction (Additional Figure S3). Many of the expanded genes were related to lipid metabolism, such as fatty acid elongation, fatty acid degradation, glycerolipid metabolism, and steroid hormone biosynthesis (Additional Figure S4). Lipid metabolism is vital for gross changes in the body shape of the female *E. pela* insect [5]. The fatty acids in lipid metabolism are key substrates for wax biosynthesis in *E. pela*.

## Potential Implications

Scale insects have considerable diversity in terms of evolutionary lineages, morphology, species richness, and genetic systems. Scales are important features of many agricultural pests and invasive species. However, the relationships between scale insect families are uncertain, despite >100 years of phylogenetic studies. Sequencing the genome of scale insects will help to show where they belong on the scale insect superfamily tree. The genomic data will also help determine the phylogenetic relationships between insects, and reveal the genomic basis of insect evolution and environmental adaptation. With the increasing availability of insect genome sequences, we gain global perspectives that enable research on the mechanisms of the life activities of insects, and the mechanisms underlying different biological characteristics. Some insect genome sequence projects, such as i5k [54] and TOP1000, will provide new insights and accelerate insect research.

## Conclusions

Here, we present the first genome for the Coccidae family of scale insects. The assembled *E. pela* genome and its evolutionary analysis are important and may provide insights into the mechanisms underlying the wax secretion trait. These data may also shed light on the evolution of the unique features of scale insects living in exposed environments. The *E. pela* genome provides essential information for important functional gene mining and for further evolutionary analysis.

## Availability of supporting data and materials

The dataset supporting the results of this article is available from the GenBank repository under accession number QBOQ00000000 and BioProject number PRJNA448657. All supporting data and materials are available in the *GigaScience* GigaDB database [55].

## Additional files

Additional Figure S1. The distribution of sequence divergence rates for transposable elements in the *Ericerus pela* genome, as predicted by *de novo* and homology-based approaches.

A. Transposable elements (TEs) in the *Ericerus pela* genome, as identified by a *de novo* approach. B. TEs in the *E. pela* genome, as identified by a homology-based approach. Abbreviations: LINE, long interspersed nuclear element; SINE, short interspersed nuclear element; LTR, long terminal repeat retrotransposons; DNA: DNA transposons.

Additional Figure S2. The phylogenetic tree of 14 arthropod species based on gene orthology

Thirteen insect species and *Hydra vulgaris* were used for the analysis. The bootstrap values are shown on the branches.

Additional Figure S3. The KEGG classification of contracted genes in *Ericerus pela*

Additional Figure S4. The KEGG classification of expanded genes in *Ericerus pela*

Additional Table S1. Summary of *Ericerus pela* sequencing data, derived from Illumina and Pacific Biosciences platforms

Additional Table S2. Genome size estimation by 17-mer analysis

Additional Table S3. Noncoding RNA in the *Ericerus pela* genome

Additional Table S4. Gene family contraction of 14 species (*P* < 0.01)

Additional Table S5. Gene family expansion of 14 species (*P* < 0.01)

giz113_GIGA-D-18-00371_Original_Submission

giz113_GIGA-D-18-00371_Revision_1

giz113_GIGA-D-18-00371_Revision_2

giz113_GIGA-D-18-00371_Revision_3

giz113_Response_to_Reviewer_Comments_Original_Submission

giz113_Response_to_Reviewer_Comments_Revision_1

giz113_Response_to_Reviewer_Comments_Revision_2

giz113_Reviewer_1_Report_Original_Submission Denis Tagu -- 11/30/2018 Reviewed

giz113_Reviewer_2_Report_Original_Submission Maarten Reijnders -- 4/5/2019 Reviewed

giz113_Reviewer_2_Report_Revision_1 Maarten Reijnders -- 7/4/2019 Reviewed

giz113_Supplemental_Files

## Abbreviations

BLAST: Basic Local Alignment Search Tool; bp: base pairs; BUSCO: Benchmarking Universal Single-Copy Orthologs; Gb: gigabase pairs; GST: glutathione S-transferase; kb: kilobase pairs; Mb: megabase pairs; MYA: million years ago; KEGG: Kyoto Encyclopedia of Genes and Genomes; NCBI: National Center for Biotechnology Information: PacBio: Pacific Biosciences; rRNA: ribosomal RNA; SOAP: short oligonucleotide alignment program; TE: transposable element; TRF: Tandem Repeats Finder; tRNA: transfer RNA.

## Competing interests

The authors declare that they have no competing interests.

## Funding

This study was financially supported by the Key Program of Fundamental Research Funds for the Chinese Academy of Forestry (grant number CAFYBB2017ZB005), the Special Fund for Forestry Research in the Public Interest (grant numbers 201504302 and 201304808), the National Natural Science Foundation of China (grant numbers 31572337 and 31000983), the National High Technology Research and Development Program (“863” Program) of China (grant number 2014AA021801), the Applied Basic Research Foundation of Yunnan Province (grant numbers 2013FA052 and 2010ZC235), and a RIRI-CAF National Nonprofit Institute Research Grant (grant numbers riricaf200904M-3, riricaf2011006M).

## Authors’ contributions

P.Y. conceived and designed the experiments. P.Y., S.Y., J.H., W.L., and Z.Zhao analyzed the data. P.Y., S.Y., J.H., Z.Zhao, T.S., and X.W. drafted the manuscript. P.Y., S.Y., W.L., Z.Zhao, T.S., and X.W. prepared the samples and collected the data. Z.Zhu and Q.S. modified the manuscript. All authors read and approved the final version of the manuscript.

## References

[1] Liu WW, Yang P, Chen XM, et al. Cloning and expression analysis of four heat shock protein genes in *Ericerus pela* (Homoptera: Coccidae). J Insect Sci. 2014;14:1–9. 25826465 10.1093/jisesa/ieu032 PMC5443611

[2] Sun T, Wang XQ, Zhao ZL, et al. A lethal fungus infects the Chinese white wax scale insect and causes dramatic changes in the host microbiota. Sci Rep. 2018;8:5324. 29593315 10.1038/s41598-018-23671-1 PMC5871785

[3] Yang P, Zhu JY, Gong ZJ, et al. Transcriptome analysis of the Chinese white wax scale *Ericerus pela* with focus on genes involved in wax biosynthesis. PLoS One. 2012;7:e35719. 22536429 10.1371/journal.pone.0035719 PMC3334986

[4] Yang P, Chen XM Protein profiles of Chinese white wax scale, *Ericerus pela*, at the male pupal stage by high-throughput proteomics. Arch Insect Biochem Physiol. 2014;87:214–33. 25186183 10.1002/arch.21191

[5] Yang P, Chen XM, Liu WW, et al. Transcriptome analysis of sexually dimorphic Chinese white wax scale insects reveals key differences in developmental programs and transcription factor expression. Sci Rep. 2015;5:8141. 25634031 10.1038/srep08141 PMC4311254

[6] Yu SH, Yang P, Sun T, et al. Transcriptomic and proteomic analyses on the supercooling ability and mining of antifreeze proteins of the Chinese white wax scale insect. Insect Sci. 2016;23:430–7. 26799455 10.1111/1744-7917.12320

[7] Yu SH, Yang P, Sun T, et al. Identification and evaluation of reference genes in the Chinese white wax scale insect *Ericerus pel*a. Springerplus. 2016;5:791. 27390632 10.1186/s40064-016-2548-z PMC4916112

[8] Chen Y, Chen X, Wang Z, et al. Studies on secreting wax of Chinese white wax scale: the comparison of secreting wax on different host plants. Forest Res. 1998;11:285–8.

[9] Morse GE, Normark BB A molecular phylogenetic study of armoured scale insects (Hemiptera: Diaspididae). Syst Entomol. 2006;31:338–49.

[10] Gullan PJ, Cook LG Phylogeny and higher classification of the scale insects (Hemiptera: Sternorrhyncha: Coccoidea). Zootaxa. 2007;1668:413–25.

[11] Hodgson CJ, Hardy NB The phylogeny of the superfamily Coccoidea (Hemiptera: Sternorrhyncha) based on the morphology of extant and extinct macropterous males. Syst Entomol. 2013;38:794–804.

[12] Wang XQ, Yu SH, Sun T, et al. Analysis of the diversity of microoraganisma in the wax secreted by the Chinese white wax scale insect, *Ericerus pela* (Chanvannes) (Homoptera: Coccidae). Acta Entomol Sin. 2016;59:1086–92.

[13] Gullan PJ, Kosztarab M Adaptations in scale insects. Annu Rev Entomol. 1997;42:23–50. 15012306 10.1146/annurev.ento.42.1.23

[14] Li R, Zhu H, Ruan J, et al. De novo assembly of human genomes with massively parallel short read sequencing. Genome Res. 2010;20:265–72. 20019144 10.1101/gr.097261.109 PMC2813482

[15] Kajitani R, Toshimoto K, Noguchi H, et al. Efficient *de novo* assembly of highly heterozygous genomes from whole-genome shotgun short reads. Genome Res. 2014;24:1384–95. 24755901 10.1101/gr.170720.113 PMC4120091

[16] Ye C, Hill CM, Wu S, et al. DBG2OLC: efficient assembly of large genomes using long erroneous reads of the third generation sequencing technologies. Sci Rep. 2016;6:31900. 27573208 10.1038/srep31900 PMC5004134

[17] Boetzer M, Henkel CV, Jansen HJ, et al. Scaffolding pre-assembled contigs using SSPACE. Bioinformatics. 2011;27:578–9. 21149342 10.1093/bioinformatics/btq683

[18] English AC, Salerno WJ, Reid JG PBHoney: identifying genomic variants via long-read discordance and interrupted mapping. BMC Bioinformatics. 2014;15:180. 24915764 10.1186/1471-2105-15-180 PMC4082283

[19] Xie W, Chen C, Yang Z, et al. Genome sequencing of the sweetpotato whitefly *Bemisia tabaci* MED/Q. Gigascience. 2017;6:1–7.. doi:10.1093/gigascience/gix018 10.1093/gigascience/gix018 PMC5467035 28327996

[20] Wang L, Tang N, Gao X, et al. Genome sequence of a rice pest, the white-backed planthopper (*Sogatella furcifera*). Gigascience. 2017;6:1–9.. doi:10.1093/gigascience/giw004 10.1093/gigascience/giw004 PMC5437944 28369349

[21] Xue J, Zhou X, Zhang CX, et al. Genomes of the rice pest brown planthopper and its endosymbionts reveal complex complementary contributions for host adaptation. Genome Biol. 2014;15:521. 25609551 10.1186/s13059-014-0521-0 PMC4269174

[22] International Aphid Genomics Consortium. Genome sequence of the pea aphid *Acyrthosiphon pisum*. PLoS Biol. 2010;8:e1000313. 20186266 10.1371/journal.pbio.1000313 PMC2826372

[23] Luo R, Liu B, Xie Y, et al. SOAPdenovo2: an empirically improved memory-efficient short-read de novo assembler. Gigascience. 2012;1:18. 23587118 10.1186/2047-217X-1-18 PMC3626529

[24] Simão FA, Waterhouse RM, Ioannidis P, et al. BUSCO: assessing genome assembly and annotation completeness with single-copy orthologs. Bioinformatics. 2015;31:3210–2. 26059717 10.1093/bioinformatics/btv351

[25] Benson G Tandem repeats finder: a program to analyze DNA sequences. Nucleic Acids Res. 1999;27:573–80. 9862982 10.1093/nar/27.2.573 PMC148217

[26] Gertz EM, Yu YK, Agarwala R, et al. Composition-based statistics and translated nucleotide searches: improving the TBLASTN module of BLAST. BMC Biol. 2006;4:41. 17156431 10.1186/1741-7007-4-41 PMC1779365

[27] Birney E, Clamp M, Durbin R GeneWise and genomewise. Genome Res. 2004;14:988–95. 15123596 10.1101/gr.1865504 PMC479130

[28] Stanke M, Morgenstern B AUGUSTUS: a web server for gene prediction in eukaryotes that allows user-defined constraints. Nucleic Acids Res. 2005;33:W465–7. 15980513 10.1093/nar/gki458 PMC1160219

[29] Pertea M, Pertea GM, Antonescu CM, et al. StringTie enables improved reconstruction of a transcriptome from RNA-seq reads. Nat Biotechnol. 2015;33:290–5. 25690850 10.1038/nbt.3122 PMC4643835

[30] Elsik CG, Mackey AJ, Reese JT, et al. Creating a honey bee consensus gene set. Genome Biol. 2007;8(1):R13. 17241472 10.1186/gb-2007-8-1-r13 PMC1839126

[31] Lowe TM, Chan PP tRNAscan-SE On-line: search and contextual analysis of transfer RNA genes. Nucleic Acids Res. 2016;44:W54–7. 27174935 10.1093/nar/gkw413 PMC4987944

[32] Griffiths-Jones S, Moxon S, Marshall M, et al. Rfam: annotating non-coding RNAs in complete genomes. Nucleic Acids Res. 2005;33:D121–4. 15608160 10.1093/nar/gki081 PMC540035

[33] Li L, Stoeckert CJ S, Roos DS OrthoMCL: identification of ortholog groups for eukaryotic genomes. Genome Res. 2003;13:2178–89. 12952885 10.1101/gr.1224503 PMC403725

[34] Guindon S, Gascuel O A simple, fast, and accurate algorithm to estimate large phylogenies by maximum likelihood. Syst Biol. 2003;52:696–704. 14530136 10.1080/10635150390235520

[35] Guindon S, Dufayard JF, Lefort V, et al. New algorithms and methods to estimate maximum-likelihood phylogenies: assessing the performance of PhyML 3.0. Syst Biol. 2010;59:307–21. 20525638 10.1093/sysbio/syq010

[36] Benton MJ, Donoghue PC Paleontological evidence to date the tree of life. Mol Biol Evol. 2007;24:26–53. 17047029 10.1093/molbev/msl150

[37] Donoghue PCJ, Benton MJ Rocks and clocks: calibrating the tree of life using fossils and molecules. Trends Ecol Evol. 2007;22:424–31. 17573149 10.1016/j.tree.2007.05.005

[38] Dunn CW, Howison M, Zapata F Agalma: an automated phylogenomics workflow. BMC Bioinformatics. 2013;14:330. 24252138 10.1186/1471-2105-14-330 PMC3840672

[39] Edgar RC Muscle: multiple sequence alignment with high accuracy and high throughput. Nucleic Acids Res. 2004;32:1792–7. 15034147 10.1093/nar/gkh340 PMC390337

[40] Rannala B, Yang Z Inferring speciation times under an episodic molecular clock. Syst Biol. 2007;56:453–66. 17558967 10.1080/10635150701420643

[41] Yang Z PAML 4: phylogenetic analysis by maximum likelihood. Mol Biol Evol. 2007;24:1586–91. 17483113 10.1093/molbev/msm088

[42] Yang Z, Rannala B Bayesian estimation of species divergence times under a molecular clock using multiple fossil calibrations with soft bounds. Mol Biol Evol. 2006;23:212–26. 16177230 10.1093/molbev/msj024

[43] Li H, Coghlan A, Ruan J, et al. TreeFam: a curated database of phylogenetic trees of animal gene families. Nucleic Acids Res. 2006;34:D572–80. 16381935 10.1093/nar/gkj118 PMC1347480

[44] Li R, Fan W, Tian G, et al. The sequence and de novo assembly of the giant panda genome. Nature. 2010;463:311–7. 20010809 10.1038/nature08696 PMC3951497

[45] De Bie T, Cristianini N, Demuth JP, et al. CAFE: a computational tool for the study of gene family evolution. Bioinformatics. 2006;22:1269–71. 16543274 10.1093/bioinformatics/btl097

[46] Pavlidi N, Khalighi M, Myridakis A, et al. A glutathione-S-transferase (TuGSTd05) associated with acaricide resistance in *Tetranychus urticae* directly metabolizes the complex II inhibitor cyflumetofen. Insect Biochem Mol Biol. 2017;80:101–15. 27932274 10.1016/j.ibmb.2016.12.003

[47] Sookrung N, Reamtong O, Poolphol R, et al. Glutathione S-transferase (GST) of American cockroach, *Periplaneta americana*: classes, isoforms, and allergenicity. Sci Rep. 2018;8:484. 29323160 10.1038/s41598-017-18759-z PMC5764987

[48] Zhao JJ, Fan DS, Zhang Y, et al. Identification and characterisation of putative glutathione S-transferase genes from *Daktulosphaira vitifoliae* (Hemiptera: Phylloxeridae). Environ Entomol. 2018;47:196–203. 29293981 10.1093/ee/nvx184

[49] Auiyawong B, Narawongsanont R, Tantitadapitak C Characterization of AKR4C15, a novel member of aldo-keto reductase, in comparison with other rice AKR(s). Protein J. 2017;36:257–69. 28699078 10.1007/s10930-017-9732-z

[50] Di Luccio E, Elling RA, Wilson DK Identification of a novel NADH-specific aldo-keto reductase using sequence and structural homologies. Biochem J. 2006;400:105–14. 16813561 10.1042/BJ20060660 PMC1635432

[51] Mochizuki S, Nishiyama R, Inoue A, et al. A novel aldo-keto reductase, HdRed, from the Pacific abalone *Haliotis discus* hannai, which reduces alginate-derived 4-deoxy-L-erythro-5-hexoseulose uronic acid to 2-keto-3-deoxy-D-gluconate. J Biol Chem. 2015;290:30962–74. 26555267 10.1074/jbc.M115.686725 PMC4692223

[52] Gene Ontology Consortium. The Gene Ontology (GO) database and informatics resource. Nucleic Acids Res. 2004;32:D258–61. 14681407 10.1093/nar/gkh036 PMC308770

[53] Kanehisa M, Araki M, Goto S, et al. KEGG for linking genomes to life and the environment. Nucleic Acids Res. 2007;D480–4. 10.1093/nar/gkm882 PMC2238879 18077471

[54] i5K Consortium. The i5K Initiative: advancing arthropod genomics for knowledge, human health, agriculture, and the environment. J Hered. 2013;104(5):595–600. 23940263 10.1093/jhered/est050 PMC4046820

[55] Yang P, Yu S, Hao J, et al. Supporting data for “Genome sequence of the Chinese white wax scale insect: the first draft genome for the Coccidae family of scale insects”. GigaScience. 2019 10.5524/100631. PMC6743827 31518402

