# Supplementary material for: Genome sequence of the Chinese white wax scale insect Ericerus pela: the first draft genome for the Coccidae family of scale insects
Source: Gigascience. 2019 Sep 13;8(9):giz113. doi: 10.1093/gigascience/giz113 (PMC6743827; doi:10.1093/gigascience/giz113)
Supplement: giz113_GIGA-D-18-00371_Original_Submission [file giz113_giga-d-18-00371_original_submission.pdf]

GigaScience

Genome Sequence of the Chinese White Wax Scale Insect: the First Draft Genome in the Coccidae Family of Scale Insects

--Manuscript Draft--

**Manuscript Number:**	GIGA-D-18-00371
**Full Title:**	Genome Sequence of the Chinese White Wax Scale Insect: the First Draft Genome in the Coccidae Family of Scale Insects
**Article Type:**	Data Note
**Funding Information:**	Key Program of Fundamental Research Funds for the Chinese Academy of Forestry (CAFYBB2017ZB005) — Dr Pu Yang<br>Special Fund for Forestry Research in the Public Interest (201504302) — Dr Pu Yang<br>Special Fund for Forestry Research in the Public Interest (201304808) — Dr Pu Yang<br>National Natural Science Foundation of China (31572337) — Dr Pu Yang<br>National Natural Science Foundation of China (31000983) — Dr Pu Yang<br>Applied Basic Research Foundation of Yunnan Province (2013FA052) — Dr Pu Yang
**Abstract:**	Background: The Chinese white wax scale insect, Ericerus pela, is best known for its important role in producing wax that has been widely used in candle production, casting, Chinese medicine, and wax printing products over thousands of years. The wax secretion and other unusual features of scale insects are likely adaptations to their change from an ancestral ground-dweller lifestyle to a subsequent sedentary lifestyle living on the higher positions of plants. In addition to aiding their economic importance, study of E. pela should also help explain the adaptation of scale insects. However, there is currently no genomic data from E. pela. Findings: To assemble the E. pela genome, a total of 303.92 Gb was sequenced using Illumina and PacBio sequencing, producing 277.22 Gb of clean data for assembly. The assembled genome size of E. pela was 0.66 Gb with 1,979 scaffolds, and a scaffold N50 of 735 kb. The G+C content was 33.80%. A total of 12,022 protein-coding genes were predicted, with an average coding sequence length of 1,370 bp. A total of 26 fatty acyl-CoA reductase genes and 35 acyltransferase genes were identified. Evolutionary analysis suggested that E. pela and aphids formed a sister group that split approximately 241.1 million years ago. There were 214 expanded gene families and 2,219 contracted gene families in E. pela. Conclusion: We present the first genome from the Coccidae family. The results increase our understanding of the evolution of unique features in scale insects and provide important genetic information for further research.
**Corresponding Author:**	Pu Yang<br><br>CHINA
**Corresponding Author Secondary Information:**	
**Corresponding Author's Institution:**	
**Corresponding Author's Secondary Institution:**	
**First Author:**	Pu Yang
**First Author Secondary Information:**	

Powered by Editorial Manager® and ProduXion Manager® from Aries Systems Corporation

**Order of Authors:**	Pu Yang
	Shuhui Yu
	Junjun Hao
	Zunling Zhao
	Zengrong Zhu
	Xueqing Wang
	Tao Sun
	Xiaoming Chen
	Qisheng Song
**Order of Authors Secondary Information:**	
**Additional Information:**	
**Question**	**Response**
Are you submitting this manuscript to a special series or article collection?	No
**Experimental design and statistics**<br><br>Full details of the experimental design and statistical methods used should be given in the Methods section, as detailed in our Minimum Standards Reporting Checklist. Information essential to interpreting the data presented should be made available in the figure legends.<br><br>Have you included all the information requested in your manuscript?	Yes
**Resources**<br><br>A description of all resources used, including antibodies, cell lines, animals and software tools, with enough information to allow them to be uniquely identified, should be included in the Methods section. Authors are strongly encouraged to cite Research Resource Identifiers (RRIDs) for antibodies, model organisms and tools, where possible.<br><br>Have you included the information requested as detailed in our Minimum Standards Reporting Checklist?	Yes

**Availability of data and materials**<br><br>All datasets and code on which the conclusions of the paper rely must be either included in your submission or deposited in publicly available repositories (where available and ethically appropriate), referencing such data using a unique identifier in the references and in the "Availability of Data and Materials" section of your manuscript.<br><br>Have you have met the above requirement as detailed in our Minimum Standards Reporting Checklist?	Yes

Powered by Editorial Manager® and ProduXion Manager® from Aries Systems Corporation

Manuscript

Click here to view linked References

Click here to access/download;Manuscript;waxscale_manuscript_Edit_finalJ

Genome Sequence of the Chinese White Wax Scale Insect: the First Draft Genome in the Coccidae Family of Scale Insects

Pu Yang[*#1], Shuhui Yu[#2], Junjun Hao[#3], Zunling Zhao[1], Zengrong Zhu[4], Xueqing Wang[1], Tao Sun[1], Xiaoming Chen[*1], Qisheng Song[5]

1. Research Institute of Resource Insects, Chinese Academy of Forestry, Key Laboratory of Cultivating and Utilization of Resources Insects of State Forestry Administration, Kunming, 650224, China

2. School of Agriculture, Kunming University, 650214, Kunming, China.

3. State Key Laboratory of Genetic Resources and Evolution, Laboratory of Evolutionary and Functional Genomics, Kunming Institute of Zoology, Chinese Academy of Sciences, Kunming, 650223, Yunnan, China

4. State Key Laboratory of Rice Biology/Key Laboratory of Agricultural Entomology Ministry of Agriculture/Institute of Insect Sciences, Zhejiang University, Hangzhou, 310058, China

5. Division of Plant Sciences, University of Missouri , 1-31 Agriculture Building, Columbia, Missouri, 65211, United States.

Pu Yang, Research Institute of Resource Insects, Kunming, 650224, China. E-mail: zjuyangpu@aliyun.com. ORCID iD: 0000-0001-9949-1265.

Shuhui Yu, Kunming University, 650214, Kunming, China. E-mail: shuhui19841015@126.com. ORCID iD: 0000-0003-0219-6564.

Junjun Hao, Kunming Institute of Zoology, Kunming, 650223, Yunnan, China. E-mail: 76918054@qq.com

Zunling Zhao, Research Institute of Resource Insects, Kunming, 650224, China. E-mail: 570912576@qq.com

Zengrong Zhu, Zhejiang University, Hangzhou, 310058, China. E-mail: zrzhu@zju.edu.cn

Xueqing Wang, Research Institute of Resource Insects, Kunming, 650224, China. E-mail: 573461228@qq.com

Tao Sun, Research Institute of Resource Insects, Kunming, 650224, China. E-mail: 814437924@qq.com

Xiaoming Chen, Research Institute of Resource Insects, Kunming, 650224, China. E-mail: 61932167@qq.com

Qisheng Song, Division of Plant Sciences, University of Missouri, Columbia, Missouri, 65211, United States. E-mail: songq@missouri.edu

#These authors contributed equally to this work.

*Correspondence: zjuyangpu@aliyun.com

Abstract

Background: The Chinese white wax scale insect, *Ericerus pela*, is best known for its important role in producing wax that has been widely used in candle production, casting, Chinese medicine, and wax printing products over thousands of years. The wax secretion and other unusual features of scale insects are likely adaptations to their change from an ancestral ground-dweller lifestyle to a subsequent sedentary lifestyle living on the higher positions of plants. In addition to aiding their economic importance, study of *E. pela* should also help explain the adaptation of scale insects. However, there is currently no genomic data from *E. pela*. **Findings**: To assemble the *E. pela* genome, a total of 303.92 Gb was sequenced using Illumina and PacBio sequencing, producing 277.22 Gb of clean data for assembly. The assembled genome size of *E. pela* was 0.66 Gb with 1,979 scaffolds, and a scaffold N50 of 735 kb. The G+C content was 33.80%. A total of 12,022 protein-coding genes were predicted, with an average coding sequence length of 1,370 bp. A total of 26 fatty acyl-CoA reductase genes and 35 acyltransferase genes were identified. Evolutionary analysis suggested that *E. pela* and aphids formed a sister group that split approximately 241.1 million years ago. There were 214 expanded gene families and 2,219 contracted gene families in *E. pela*. **Conclusion**: We present the first genome from the Coccidae family. The results increase our understanding of the evolution of unique features in scale insects and provide important genetic information for further research.

Key words: *Ericerus pela*, Chinese white wax scale insect, wax secretion, adaptation, genome.

Data Description

The Chinese white wax scale insect (*Ericerus pela*), silkworm (*Bombyx mori*), and honeybee (*Apis cerana*) are the three traditionally domesticated insect species of China. *E. pela* (NCBI: txid931557) is best-known for its important role in Chinese wax production (Fig. 1). With the discovery of the useful properties of this white insect wax, it has been harvested for candles and polishes, as well as the food, medicine, and cosmetics industries in China and Japan [1-7]. Insect wax materials are derived from white wax (produced by *E. pela*) and yellow wax (produced by *A. cerana*). However, *E. pela* is the main wax producer. Each individual *E. pela* produces on average approximately 0.45 mg of wax on the host glossy privet (*Ligustrum lucidum*) tree [8]. Annual amounts of wax production range from 300 to 500 tons, and this produces revenue of approximately 60 to 100 million Yuan in China. The long-chain alcohols made from white wax and other white wax products have additional economic value.

E. pela is a typical scale insect, of which wax secretion is their most striking feature. There are two major groups (archaeococcoids and neococcoids) and approximately 8,000 species of scale insects. The neococcoids, which are the more recent forms, include 17 families, such as Coccidae, Pseudococcidae, and Dactylopiidae. Some of these species are important pets or resource insects [9-11]. *E. pela* belongs to the family Coccidae and genus *Ericerus*, and it is the only species in the genus *Ericerus*. The “scale” in their name derives from the common protective cover formed by wax

secretions on this type of insect. To serve protective functions, the wax secretions have antimicrobial activity and hydrophobic properties, so the secretions of a few scale insects have been applied in the industrial field [2, 12]. In the husbandry process, insects are fed and deposit their secretions on the branches of certain species of *Ligustrum* (privet) trees, and these are harvested and boiled with water to extract the raw wax. At the end of the process, the leftover insect bodies are used as animal feed.

Before the diversification of angiosperms, ancestral scale insects were initially ground leaf litter dwellers. Many special features of scale insects are legacies of adaptations to this ancestral lifestyle. With the increasing predominance of seed plants, scale insects have transferred to the aerial part of seed plants. This exposed living environment and predation have placed selective pressure on scale insects, meaning their protective cover enhances the survival success of these insects. Wax secretion is a special survival strategy adapted to a sedentary lifestyle on host plants [12, 13]. Apart from wax secretion, the best known feature of scale insects is possibly their sexual dimorphism. The females reduce or lose appendages, and their body shape appears as a sphere. Males and females are sexually dimorphic in many aspects and appear to be two different species [1, 4, 5]. Sexual dimorphism is beneficial to male courtship and female reproduction in *E. pela* and makes full use of the resources within a habitat. *E. pela* is a typical scale insect and provides opportunities for investigating the wax secretion mechanism and adaptive evolution of scale insects under specific environments.

We previously studied the molecular biology of white wax biosynthesis, sexual

dimorphism, antifreeze biology, and microbial symbiosis using transcriptome and gene expression profiles and gene cloning and expression techniques [1-7, 12]. However, there are currently no genomic data for *E. pela*, which hinders obtaining a deeper understanding of the biology and genetics of *E. pela*. In this study, we constructed seven libraries of different insert sizes for Illumina and PacBio sequencing and assembled the *E. pela* genome. This information will aid in breeding and variety selection of this species and will be useful in species conservation. In addition, the genome will provide insight into phylogenetic relationships of *E. pela* with other insects in the tree of life and the relationships among the families of scale insects. The data provide information for insect systematics and evolution research and could fill a gap in some projects for phylogenetic relationship research and the genomic basis of insect diversity analyses.

Sample preparation and library construction

E. pela individuals from the Kunming geographical population were reared in the Research Institute of Resource Insects, Kunming, China. Each individual can produce thousands of offspring. The *E. pela* offspring produced by one individual were reared on one host tree planted in one flower pot. To remove the microbial symbionts, the female adults were washed 3 times in ddH_2O for 5 min and then dissected in phosphate buffered saline (PBS, pH=7.4) under a stereomicroscope. The cuticle, gut, and ovaries were then detached carefully, and the remaining tissue was washed 3 times in cold PBS for 5 min. Twenty individuals were used for genomic DNA isolation.

The samples were crushed to powder in a mortar with liquid nitrogen. Then, 3 mL of lysis buffer (10 mM Tris-HCl, 400 mM NaCl, 2 mM EDTA-2Na, 0.8 M guanidine hydrochloride), 20 μL of proteinase K (50 mg/mL) and 200 μL of SDS were added. The solution was incubated at 56°C for 45 min. Next, 3.5 mL of isolation buffer (240 mL chloroform, 10 mL isoamyl alcohol, 250 mL Tris-phenol) was added to the solution before centrifugation at 4,700 rpm for 10 min. The supernatant was transferred to a new tube, and the isolation step was repeated. Then, 3 mL of isopropyl alcohol (precooled to -20°C) was added to the supernatant. The DNA precipitate was obtained and washed with 70% ethanol, and then, 100 μL of TE was added to dissolve the DNA after the ethanol volatilized completely. To degrade the RNA, 2 μL of RNase A (10 mg/mL) was added. The DNA concentration was determined with a Nanodrop 8000 (Thermo Scientific, U.S.) and a Qubit Fluorometer (Invitrogen, U.S.). The DNA quality was tested by pulsed-field gel electrophoresis.

A total of 6 libraries with a gradient of insert sizes (200 bp, 350 bp, 500 bp, 2 kb, 5 kb, and 10 kb) (Supplementary Table S1) were constructed for second-generation sequencing. For each of the three small insert size libraries, 2 μg of genomic DNA (concentration ≧ 20 ng/ul) was broken into 200 bp, 350 bp, or 500 bp fragments separately by an ultrasonic processor. After end repair, A-tail addition, sequence adaptor addition, purification, and PCR, the libraries were constructed according to the manufacturer’s protocol (Illumina, San Diego, CA, US). For the 2 kb, 5 kb, and 10 kb libraries, approximately 20 μg of genomic DNA was fragmented by an ultrasonic processor to construct each library. After end repair, the fragments were biotinylated.

Target fragments were selected on an agarose gel. To capture DNA fragments that circularized by self-ligation, the DNA was fragmented again and biotinylated. After purification with M-280 streptavidin Dynabeads (Invitrogen, U.S.), the fragments were end-repaired, the A-tail was added, and the adaptor was ligated to the fragments. PCR amplification was performed, and 400-600 bp products were selected on an agarose gel and purified. The library was then quantitated with a Qubit 2.0 and diluted to 1.5 ng/μL. The insert size of the libraries was detected on an Agilent 2100 bioanalyzer (Agilent, CA, U.S.). Real-time quantitative PCR was performed to quantify the libraries. Then, the libraries were sequenced on an Illumina HiSeq 2500 or HiSeq 2000 system (Illumina, San Diego, CA, U.S.).

A PacBio 20K library was constructed for the third-generation sequencing. Approximately 10 μg of genomic DNA was broken into fragments of approximately 17 kb. The fragments were digested by ExoVII, damage repaired, and end repaired. The fragments were then ligated overnight with adaptors. After enzyme digestion and fragment size selection, the library was constructed. The templates were annealed with primers, subjected to bind polymerase and sequenced on a PacBio Sequel system (Menlo Park, CA, U.S.) using the MagBead loading model.

Data processing, genome evaluation and assembly

The sequence quality was assessed with sequence quality distribution, error rate distribution, and GC content analysis. The raw data were filtered as follows: (1) adaptor sequences were removed; (2) when the N content in the reads obtained from single-end sequencing was above 10%, the paired reads were removed; and (3) when

the content of low-quality bases in the reads obtained from single-end sequencing was above 50%, the paired reads were removed.

Error correction was performed to correct the filtered data of the 3 libraries with small inserts. Sequence errors can result in new k-mers with low frequencies. A k-mer frequency of 10 was considered the cutoff between low and high frequencies for error correction. Some bases in the reads that had a low frequency were corrected to generate a higher frequency [14].

The k-mers method [14] was used to examine the *E. pela* genome size and heterozygosity before genome assembly. To generate a 17-mer depth frequency curve, 25,370,340,375 bp of high-quality data were used (Fig. 2). There was one peak in the curve, and the main peak was approximately 28 bp. The total k-mer number was 22,122,936,807. The genome size was calculated to be 0.79 Gb according to the following formula: Genome Size = k-mer_num/Peak _depth (Supplementary Table S2) [14]. There was no heterozygosity peak in the *E. pela* genome (Fig. 2).

The filtered data were first assembled into contigs using the Platanus software (1.2.1) [15]. Then, the contigs and the PacBio sequence data were used to assemble scaffolds with the DBG2OLC method [16]. A total of 247 Gb of second-generation data and 30 Gb of third-generation data were used for assembly. Due to the high error rate of PacBio sequencing, the scaffolds had many minor errors. Error correction was performed using the second-generation sequencing data and third-generation sequencing data. Preliminary corrections were conducted by the Pilon-1.22 software based on the alignment of the second-generation sequencing data with the assembled

sequences. After that, the scaffold was further constructed using the SSPACE software [17]. Then, the PacBio sequence data were used to fill the holes in the scaffold using the PBjelly program [18]. Finally, the Polish software was used to perform the second error correction.

The *E. pela* genome was finally assembled into 0.66 Gb. The genome consisted of 1,979 scaffolds. The N50 of the scaffolds was 735,622 bp, and the N50 of the contigs was 660,240 bp (Table 1). The genome size was similar to that of *Bemisia tabaci* (658 Mb) [19] and *Sogatella furcifera* (720 Mb) [20], smaller than that of *Nilaparvata lugens* (1,141 Mb) [21] and larger than that of *Acyrthosiphon pisum* (464 Mb) [22].

The G+C content of the *E. pela* genome was 33.80%, which is similar to that of *N. lugens* (34.60%) and *S. furcifera* (31.60%). However, it is lower than that of *B. tabaci* (39.00%) and higher than that of *A. pisum* (29.60%).

Genome assembly analysis

After genome assembly, the sequencing depth was calculated by SOAP coverage 2.27 [23]. Four transcriptome data sequences [3-6] were used as query sequences and mapped to the assembled genome sequence. The coverage of assembled sequences to transcriptome sequences was tested. The BUSCO software (version 3. http://busco.ezlab.org/) [24] was used to evaluate coding gene completeness.

The reads from four transcriptomes were mapped to the *E. pela* genome. The results showed that 88.41%, 86.67%, 86.26%, and 91.03% of the reads from the four transcriptomes could be mapped to the genome.

Repeated sequence annotation

Tandem repeat sequences were identified using the TRF software [25]. The interspersed repeat sequences (transposons) were identified using the RepeatMasker and RepeatProteinMask software based on the RepBase database. *De novo* prediction was performed using the RepeatMasker software, which is based on the database from the RepeatModeler software. Non-redundant results were obtained after all of the results predicted above were combined and overlapping results were removed.

Repeated sequences composed 55.06% of the *E. pela* genome (Table 2), which is a higher proportion than that in *N. lugens* (48.6%) [21] and *A. pisum* (33.3%) [22].

The *E. pela* transposable elements (TEs) identified through *de novo* prediction showed a shift in peak sequence compared with that identified through a homology-based approach (Fig. S1). This result suggests the recent evolution of DNA transposons, similar to the genome of *N. lugens* [21].

Gene prediction and annotation

Protein homology-based gene prediction (eight closely related species were selected) was performed using BLASTN [26]. The Augustus software [27] was used for *de novo* prediction. Then, the predicted genes from above were integrated into one non-redundant and more complete gene set using the GLEAN software [28]. In addition, the transcripts were used to supplement the gene sets after the RNA-seq data alignment (TopHat) and assembly (Cufflinks software) [29]. Finally, protein databases (SwissProt, TrEMBL, Kyoto Encyclopedia of Genes and Genomes (KEGG), InterPro, and Gene Ontology (GO)) were used to annotate the function of proteins encoded by the gene sets.

A total of 12,022 protein-coding genes were predicted using a combination of *de novo*, GLEAN, and homolog prediction (Table 3). The number of predicted genes in *E. pela* was lower than the gene numbers in several published hemipteran insects, such as *N. lugens* (27,571) [21], A. *pisum* (33,267) [22], *S. furcifera* (21,254) [20], and *B. tabaci* (20,786) [19]. A total of 87.99% of gene sets were functionally annotated (Table 4).

The average code sequence (CDS) length of *E. pela* was 1,370 bp, which is slightly longer than that of *N. lugens* (1,135 bp) [21] and shorter than that of *S. furcifera* (1,577 bp) [20]. The average intron length was 1,673 bp.

The fatty acyl-CoA reductase gene (*far*) and acyltransferase gene are related to wax secretion [3]. There were a total of 26 *far* genes in the *E. pela* genome, which is a moderate number compared to other Hemiptera insects (*A. pisum* 34; *D. noxia* 27; *D. citri* 46; *B. tabaci* 25). However, a total of 35 acyltransferase gene family members were identified in the *E. pela* genome, which is greater than that in the other 4 hemipteran insects (*A. pisum* 15; *D. noxia* 13; *D. citri* 16; *B. tabaci* 25).

Noncoding RNA annotation

tRNA was identified according to structure with the tRNAscan-SE software [30]. rRNA was identified by BLASTN alignment using the rRNA sequences from closely related species as query sequences. miRNA and snRNA were predicted using the INFERNAL software in Rfam according to the covariance model of Rfam [31]. Noncoding RNA including rRNA, tRNA, nRNA, and miRNA was identified in the *E. pela* genome (Supplementary Table S3).

Gene phylogenomics

The gene sets of 14 species (Supplementary Table S4) were filtered to obtain high-quality gene sets. Gene clusters were identified with the OrthoMCL software [32]. Single-copy and multiple-copy gene families were obtained through the identification of homologs and gene family cluster analysis. A total of 65 single-copy gene families were identified across the 14 species (Supplementary Table S4, S5).

The single-copy gene families were arrayed as a super gene after multiple-sequence alignment. This super gene was used to construct a phylogenetic tree [33, 34]. The species division time was calculated according to the molecular clock based on the four-fold degenerate codon of single-copy gene families [35-41]. The phylogenetic tree constructed based on the single-copy orthologs showed that *E. pela* was a sister group of aphids (*A. pisum* and *Diuraphis noxia*), and this group clustered together to form a sister group with psyllid (*Diaphorina citri*) (Supplementary Fig. S2). These results suggest that scale insects and aphids evolved more recently than did other hemipteran insects, such as white flies and plant lice.

The divergence time between *E. pela* and aphids was estimated to be 241.1 million years ago (mya) (Fig. 3). Fossils have shown that the ancestors of scale insects had reached modern morphology by the Lower Cretaceous period (65-137 mya). The evolution of scale insects may have occurred even earlier during the mid-Mesozoic period (60-250 mya) or earlier. Ancestral scale insects originally lived in the leaf litter layer and sucked plant roots, similar to modern thrips [13]. Angiosperms diversified until 90-130 mya, so the ancient scale insects must have fed on lower plants or fungi.

Many unique features of scale insects, such as wax secretion and appendage reduction, are considered historical legacies from their ground-dwelling ancestors. The phylogenetic tree indicated that *E. pela* diverged with aphids approximately 241.1 mya. This time supports the early evolution of scale insects and provides a reference for the ideas that the specializations of ancestral scale insects led to myriad unusual features and that the subsequent parasitic lifestyle on higher plants further favored appendage reduction and wax secretion.

The TreeFam methodology [42] was used to define a gene family that comprises a group of genes descended from one gene of the latest common ancestor [43]. The CAFÉ software was used to identify gene family expansion and contraction [44]. There were 214 expanded gene families and 2,219 contracted gene families in *E. pela* (Fig. 4). There were two gene families with gene members completely absent in the *E. pela* genome (Supplementary Table S6). The two absent gene families in the *E. pela* genome were related to RNA-directed DNA polymerase from mobile element jockey-like and glutathione S-transferases (GSTs). GSTs are thought to be important in stress response and insecticide/drug resistance [45-47]. The gene family contraction of GSTs in *E. pela* may be due to natural selection resulting from the protective function of the white wax layer. In addition, we found one gene family with gene members almost unique to *E. pela* (Supplementary Table S7). The new gene family of *E. plea* was related to aldo-keto reductases (AKRs). AKRs reduce a wide variety of carbonyl-containing compounds to corresponding alcohols in reactions dependent on NADPH [48-50]. Alcohols are one of the two substrates used to form wax esters.

The expanded and contracted gene families were selected to obtain further functional statistics. The Blast2GO and BLAST programs were used to perform GO [51] and KEGG orthology (KO) analyses [52]. GO analysis showed that the contracted genes were mainly involved in microtubule-based movement, movement of cell or subcellular components, etc. They are thought to be responsible for the sedentary lifestyle of *E. pela* on plants. The expanded genes were mainly related to nucleic acid binding, organic cyclic compound binding, and protein dimerization activity. KEGG analysis indicated that some contracted genes were related to cardiac muscle contraction (Supplementary Fig. S3). Many of the expanded genes were related to lipid metabolism, such as fatty acid elongation, fatty acid degradation, glycerolipid metabolism, and steroid hormone biosynthesis (Supplementary Fig. S4). Lipid metabolism is vital for the gross changes in female *E. pela* body shape [5]. More importantly, the fatty acids in lipid metabolism are key substrates for wax biosynthesis in *E. pela*.

Scale insects have significant diversity in evolutionary lineages, morphology, species richness, and genetic systems. Many scales are important agricultural pests and invasive species. However, the relationships among scale insect families were uncertain despite more than 100 years of phylogenetic studies. The genome sequence of scale insects will show where they belong on the scale insect superfamily tree. The genome data will also help determine the phylogenetic relationships of insects and reveal the genomic base of insect evolution and environmental adaptation. With increasing availability of insect genome sequences, we gain global perspectives to

research the laws of life activities of insects and the mechanisms underlying different biological characteristics. Some insect genome sequence projects, such as i5k and TOP1000, will provide new perspectives and accelerate insect research. Here, we present the first genome in the Coccidae family of scale insects. The assembled *E. pela* genome and evolutionary analysis provide important information and may provide insight into the mechanism underlying the wax secretion peculiarity. They may also help in understanding the evolution of unique features of scale insects living in exposed environments. The *E. pela* genome provides essential information for important functional gene mining and for further evolutionary analysis.

Availability of supporting data

The data set supporting the results of this article is available in the GenBank repository under accession number QBOQ00000000. The data were also deposit in Giga DB.

Additional files

Supplementary Fig. S1. The distribution of sequence divergence rates for TEs in the *E. plea* genome predicted by the *de novo* and homology-based approach. A. TEs in *E. plea* genome identified by the *de novo* approach. B. TEs in *E. plea* genome identified by the homology-based approach.

Supplementary Fig. S2. The phylogenetic tree of 14 arthropod species based on gene orthology.

Supplementary Fig. S3. The KEGG classification of contracted genes in *E. pela*.

Supplementary Fig. S4. The KEGG classification of expanded genes in *E. pela*.

Supplementary Table S1. Summary statistics of *E. pela* sequencing data derived from the Illumina and PacBio platforms.

Supplementary Table S2. Statistics of genome size estimation by 17-mer analysis.

Supplementary Table S3. Noncoding RNA in the *E. pela* genome.

Supplementary Table S4. The gene families in the 14 species

Supplementary Table S5. The homologous genes in the 14 species

Supplementary Table S6. Gene family contraction of the 14 species (p<0.01).

Supplementary Table S7. Gene family expansion of the 14 species (p<0.01).

Competing Interests

The authors declare that they have no competing interests.

Abbreviations

AKRs: aldo-keto reductases; CDS: code sequence; Far: fatty acyl-CoA reductase; GO: gene ontology; GSTs: glutathione S-transferases; KEGG: Kyoto encyclopedia of genes and genomes; KO: KEGG orthology; Mya: million years ago; PBS: phosphate-buffered saline; TEs: transposable elements.

Funding

This study was financially supported by the Key Program of Fundamental Research

Funds for the Chinese Academy of Forestry (CAFYBB2017ZB005), the Special Fund for Forestry Research in the Public Interest (201504302, 201304808, 201204602), the National Natural Science Foundation of China (Grant No. 31572337, 31000983), the National High Technology Research and Development Program ("863" Program) of China (2014AA021801), the Applied Basic Research Foundation of Yunnan Province (Grant No. 2013FA052, 2010ZC235), and a RIRI-CAF National Nonprofit Institute Research Grant (Grant No. riricaf200904M-3, riricaf2011006M).

Authors' Contributions

PY conceived and designed the experiments; PY, SY, JH, ZZ analyzed the data; PY, SY, JH, ZZ, XW, TS, XC drafted the manuscript; PY, SY, ZZ, XW, TS performed sample preparation and collected data; and ZZ and QS edited the manuscript.

Reference

1. Liu WW, Yang P, Chen XM, et al. Cloning and expression analysis of four heat shock protein genes in *Ericerus pela* (Homoptera: Coccidae). J Insect Sci 2014; 14: 1-9.

2. Sun T, Wang X, Zhao Z, et al. A lethal fungus infects the Chinese white wax scale insect and causes dramatic changes in the host microbiota. Sci Rep 2018; 8: 5324.

3. Yang P, Zhu JY, Gong ZJ, et al. Transcriptome analysis of the Chinese white wax scale *Ericerus pela* with focus on genes involved in wax biosynthesis. PLoS One 2012; 7: e35719.

4. Yang P, Chen XM. Protein profiles of Chinese white wax scale, *Ericerus pela*, at the male pupal stage by high-throughput proteomics. Arch Insect Biochem Physiol 2014; 87: 214-233.

5. Yang P, Chen XM, Liu WW, et al. Transcriptome analysis of sexually dimorphic Chinese white wax scale insects reveals key differences in developmental programs and transcription factor expression. Sci Rep 2015; 5: 8141.

6. Yu SH, Yang P, Sun T, et al. Transcriptomic and proteomic analyses on the supercooling ability and mining of antifreeze proteins of the Chinese white wax scale insect. Insect Sci 2016; 23: 430-437.

7. Yu SH, Yang P, Sun T, et al. Identification and evaluation of reference genes in the Chinese white wax scale insect *Ericerus pel*a. Springerplus 2016; 5: 791.

8. Chen Y, Chen X, Wang Z, et al. Studies on Secreting Wax of Chinese white wax scale: the

comparison of secreting wax on different host plants. Forest Res 1998; 11: 285-288.

9. GE Morse, BB Normark. A molecular phylogenetic study of armoured scale insects (Hemiptera: Diaspididae). Syst Entomol 2006; 31: 338–349.

10. Gullan PJ, Cook LG. Phylogeny and higher classification of the scale insects (Hemiptera: Sternorrhyncha: Coccoidea). Zootaxa 2007; 1668: 413–425.

11. Hodgson CJ, Hardy NB. The phylogeny of the superfamily Coccoidea (Hemiptera: Sternorrhyncha) based on the morphology of extant and extinct macropterous males. Syst Entomol 2013; 38: 794–804.

12. Wang XQ, Yu SH, Sun T, et al. Analysis of the diversity of microoraganisma in the wax secreted by the Chinese white wax scale insect, *Ericerus pela* (Chanvannes) (Homoptera: Coccidae). Acta Entomol Sin 2016; 59: 1086-1092.

13. Gullan PJ, Kosztarab, M. Adaptations in Scale Insects. Annu Rev Entomol 1997; 42: 23.

14. Li R, Zhu H, Ruan J, et al. De novo assembly of human genomes with massively parallel short read sequencing. Genome Res 2010; 20: 265-272.

15. Kajitani R, Toshimoto K, Noguchi H, et al. Efficient *de novo* assembly of highly heterozygous genomes from whole-genome shotgun short reads. Genome Research 2014; 24: 1384-1395.

16. Ye C, Hill CM, Wu S, et al. DBG2OLC: efficient assembly of large genomes using long erroneous reads of the third generation sequencing technologies. Sci Rep 2016; 6:31900.

17. Boetzer M, Henkel CV, Jansen HJ, et al. Scaffolding pre-assembled contigs using SSPACE. Bioinformatics 2011; 27: 578-579.

18. English AC, Salerno WJ, Reid JG. PBHoney: identifying genomic variants via long-read discordance and interrupted mapping. BMC Bioinformatics 2014; 15:180.

19. Xie W, Chen C, Yang Z, et al. Genome sequencing of the sweetpotato whitefly *Bemisia tabaci* MED/Q. Gigascience 2017; 6: 1-7.

20. Wang L, Tang N, Gao X et al. Genome sequence of a rice pest, the white-backed planthopper (*Sogatella furcifera*). Gigascience 2017; 6: 1-9.

21. Xue J, Zhou X, Zhang CX, et al. Genomes of the rice pest brown planthopper and its endosymbionts reveal complex complementary contributions for host adaptation. Genome Biol 2014; 15: 521.

22. International Aphid Genomics Consortium. Genome sequence of the pea aphid *Acyrthosiphon pisum*. PLoS Biol 2010; 8: e1000313.

23. Luo R, Liu B, Xie Y, et al. SOAPdenovo2: an empirically improved memory-efficient short-read de novo assembler. GigaScience 2012; 1: 19.

24. Simão FA, Waterhouse RM, Ioannidis P, et al. BUSCO: assessing genome assembly and annotation completeness with single-copy orthologs. Bioinformatics 2015; 31: 3210-3212.

25. Benson G. Tandem repeats finder: a program to analyze DNA sequences. Nucleic Acids Res 1999; 27: 573-580.

26. Gertz EM, Yu YK, Agarwala R, et al. Composition-based statistics and translated nucleotide searches: improving the TBLASTN module of BLAST. BMC Biology 2006; 4: 41.

27. Stanke M, Morgenstern B. AUGUSTUS: a web server for gene prediction in eukaryotes that allows user-defined constraints. Nucleic Acids Res 2005; 33: W465-467.

28. Elsik CG, Mackey AJ, Reese JT, et al. Creating a honey bee consensus gene set. Genome Biol. 2007; 8(1): R13.

29. Trapnell C, Williams BA, Pertea G, et al. Transcript assembly and quantification by RNA-Seq

reveals unannotated transcripts and isoform switching during cell differentiation. Nature Biotechnol 2010; 28: 511-515.

30. Lowe TM, Chan PP. tRNAscan-SE On-line: Search and Contextual Analysis of Transfer RNA Genes. Nucl Acids Res 2016; 44: W54-57.

31. Griffiths-Jones S, Moxon S, Marshall M, et al. Rfam: annotating non-coding RNAs in complete genomes. Nucleic Acids Research 2005; 33: D121-124.

32. Li L, Stoeckert CJ S, Roos DS. OrthoMCL: identification of ortholog groups for eukaryotic genomes. Genome Research 2003; 13: 2178-2189.

33. Guindon S, Gascuel O. A simple, fast, and accurate algorithm to estimate large phylogenies by maximum likelihood. Syst Biol 2003; 52: 696–704.

34. Guindon S, Dufayard JF, Lefort V, et al. New algorithms and methods to estimate maximum-likelihood phylogenies: assessing the performance of PhyML 3.0. Syst Biol 2010; 59: 307–321.

35. Benton MJ, Donoghue PC. Paleontological evidence to date the tree of life. Mol Biol Evol 2007; 24: 26–53.

36. Donoghue PCJ, Benton MJ. Rocks and clocks: calibrating the tree of life using fossils and molecules. Trends Ecol Evol 2007*;* 22: 424–431.

37. Dunn CW, Howison M, Zapata F. Agalma: an automated phylogenomics workflow. BMC Bioinformatics 2013; 14: 330.

38. Edgar RC. Muscle: multiple sequence alignment with high accuracy and high throughput. Nucleic Acids Research 2004; 32: 1792-1797.

39. Rannala B, Yang Z. Inferring speciation times under an episodic molecular clock. Syst Biol 2007; 56: 453–466.

40. Yang Z. PAML 4: phylogenetic analysis by maximum likelihood. Mol Biol Evol 2007; 24: 1586-1591.

41. Yang Z, Rannala B. Bayesian estimation of species divergence times under a molecular clock using multiple fossil calibrations with soft bounds. Mol Biol Evol 2006; 23: 212–226.

42. Li H, Coghlan A, Ruan J, et al. TreeFam: a curated database of phylogenetic trees of animal gene families. Nucleic Acids Res 2006; 34: D572-580.

43. Li R, Fan W, Tian G, et al. The sequence and de novo assembly of the giant panda genome. Nature 2010; 463: 311-317.

44. De Bie T, Cristianini N, Demuth JP, et al. CAFE: a computational tool for the study of gene family evolution. Bioinformatics 2006; 22: 1269-1271.

45. Pavlidi N, Khalighi M, Myridakis A, et al. A glutathione-S-transferase (TuGSTd05) associated with acaricide resistance in Tetranychus urticae directly metabolizes the complex II inhibitor cyflumetofen. Insect Biochem Mol Biol 2017; 80: 101-115.

46. Sookrung N, Reamtong O, Poolphol R, et al. Glutathione S-transferase (GST) of American cockroach, *Periplaneta americana*: classes, isoforms, and allergenicity. Sci Rep 2018; 8: 484.

47. Zhao JJ, Fan DS, Zhang Y, et al. Identification and Characterisation of Putative Glutathione S-Transferase Genes from *Daktulosphaira vitifoliae* (Hemiptera: Phylloxeridae). Environ Entomol 2018; 47: 196-203.

48. Auiyawong B, Narawongsanont R, Tantitadapitak C. Characterization of AKR4C15, a novel member of aldo-keto reductase, in comparison with other rice AKR(s). Protein J 2017; 36: 257-269.

49. Di Luccio E, Elling RA, Wilson DK. Identification of a novel NADH-specific aldo-keto reductase using sequence and structural homologies. Biochem J 2006; 400: 105-114.
50. Mochizuki S, Nishiyama R, Inoue A, et al. Ojima T. A Novel Aldo-Keto Reductase, HdRed, from the Pacific Abalone Haliotis discus hannai, Which Reduces Alginate-derived 4-Deoxy-L-erythro-5-hexoseulose Uronic Acid to 2-Keto-3-deoxy-D-gluconate. J Biol Chem 2015; 290: 30962-30974.
51. Harris MA, Clark J, Ireland A et al. The Gene Ontology (GO) database and informatics resource. Nucleic Acids Research 2004; 32: D258-261.
52. Kanehisa M, Araki M, Goto S, et al. KEGG for linking genomes to life and the environment. Nucleic Acids Res 2007; D480- D484.

Tables

Table 1. *E. pela* Genome assembly.

	Contig		Scaffold	
Type	Size (bp)	Number	Size (bp)	Number
N90	146,803	1,066	160,747	964
N80	279,168	744	309,099	673
N70	420,664	554	455,420	502
N60	530,834	414	594,019	375
N50	660,240	302	735,622	275
Maximum length	4,102,106		4,102,106	
Total length	660,732,850		660,870,788	
Total Number (≥100 bp)		2,173		1,979
Total Number (≥2 kbp)		2,168		1,979

Table 2. TEs content in the *E. pela* genome.

	Repbase TEs		TE proteins		de novo		Combined TEs	
Type	Length (bp)	% in genome	Length (bp)	% in genome	Length (bp)	% in genome	Length (bp)	% in genome
DNA	6,231,823	0.9430	1,231,066	0.1863	34,058,034	5.1535	38,499,918	5.8256
LINE	1,632,517	0.2470	98,552	0.0149	2,995,876	0.4533	4,607,151	0.6971
SINE	19,839	0.0030	0	0.0000	1,476,926	0.2235	1,491,578	0.2257
LTR	26,102,271	3.9497	53,919,001	8.1588	130,662,217	19.7712	141,132,560	21.3555
Other	15,893	0.0024	0	0.0000	0	0.0000	15,893	0.0024
Unknown	0	0.0000	0	0.0000	197,674,432	29.9112	197,674,432	29.9112
Total	32,010,886	4.8437	55,246,441	8.3596	359,818,854	54.4462	363,874,741	55.0599

Table 3. Gene predictions in the *E. pela* genome.

Gene set	Number	Average gene length (bp)	Average CDS length (bp)	Average exon per gene	Average exon length (bp)	Average intron length (bp)
augustus	19,941	11980.77	1217.04	4.78	254.54	2846.49
1-Acyrthosiphon_pisum	13,934	6838.12	1015.03	4.15	244.87	1851.39
2-Apis_mellifera	8,730	9751.43	1109.16	5.32	208.46	2000.18
3-Bemisia_tabaci	11,858	8700.69	1135.00	4.80	236.53	1991.70
4-Bombyx_mori	10,286	7557.22	988.98	4.32	228.95	1978.59
5-Drosophila_melanogaster	7,918	8712.26	1027.71	4.97	206.76	1935.38
6-Nasonia_vitripennis	12,180	6802.98	1008.16	4.14	243.53	1845.64
7-Pediculus_humanus	8,894	8903.51	1066.92	5.14	207.54	1892.54
8-Tribolium_castaneum	10,627	8850.24	1112.31	4.80	231.9	2038.15

MAKER	8,585	26163.49	1698.57	8.12	209.14	2745.54
Final	12,022	12134.44	1370.76	5.85	234.18	1673.00

Table 4. Statistics of functional annotations in the *E. pela* genome.

	Number	Percentage
Total	12,022	100.00%
Nr-Annotated	9,176	76.33%
Nt-Annotated	10,255	85.30%
Swissprot-Annotated	8,536	71.00%
KEGG-Annotated	8,628	71.77%
COG-Annotated	4,398	36.58%
TrEMBL-Annotated	10,320	85.84%
Interpro-Annotated	9,609	79.93%
GO-Annotated	3,875	32.23%
Overall	10,578	87.99%

Figure Legends

Figure 1. Branches of a Chinese glossy privet tree covered by the white wax layer secreted by *E. pela*.

Figure 2. The read distribution obtained from the 17-mer analysis.

Figure 3. The estimated time of divergence among the 14 different arthropod species.

Figure 4. The phylogenetic tree showing gene family contraction and expansion in *E. pela* compared with 13 other species.

Fig 1

Click here to access/download;Figure;Fig 1.tif

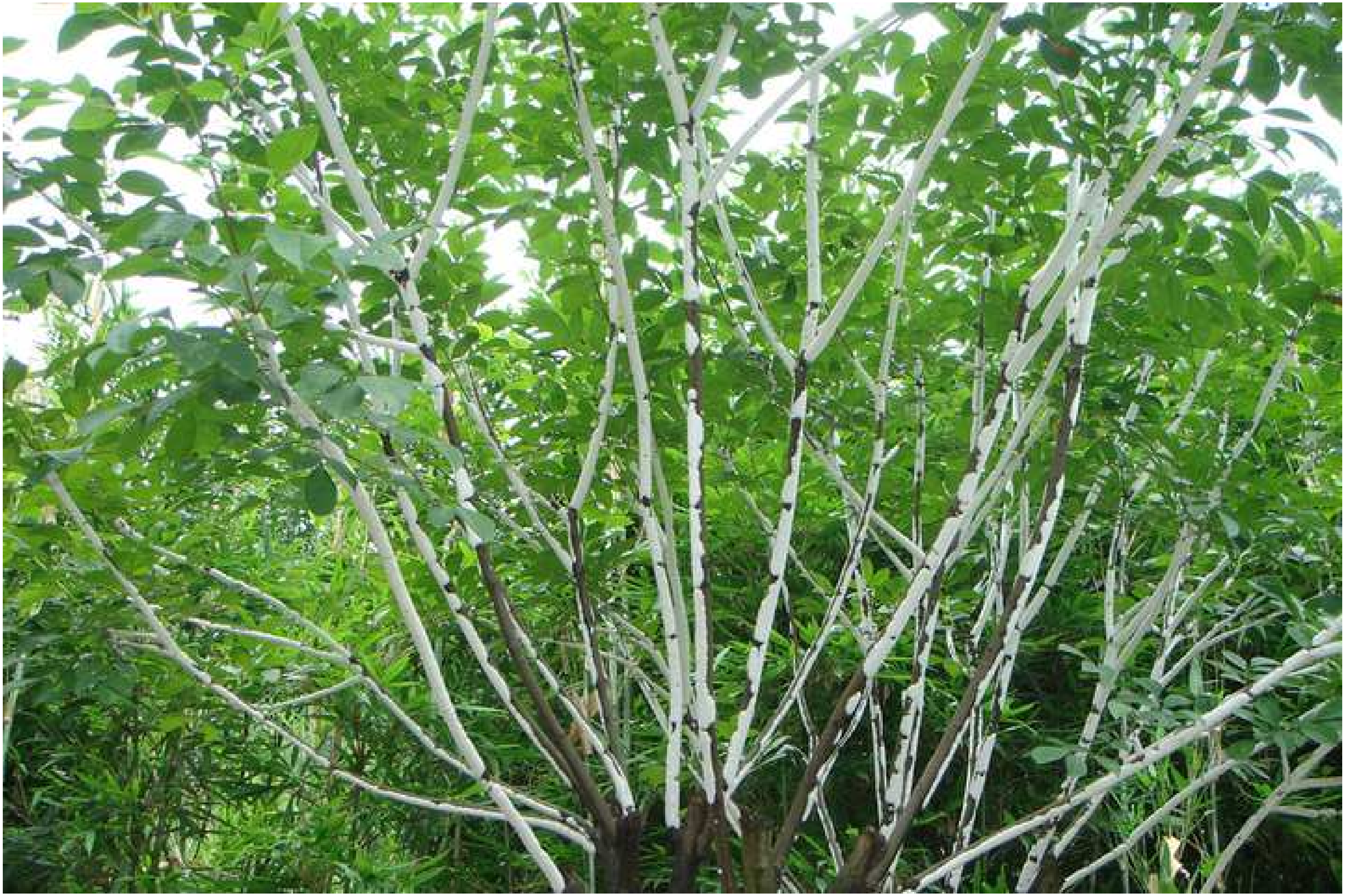

Fig 2

Click here to access/download;Figure;Fig 2.tif

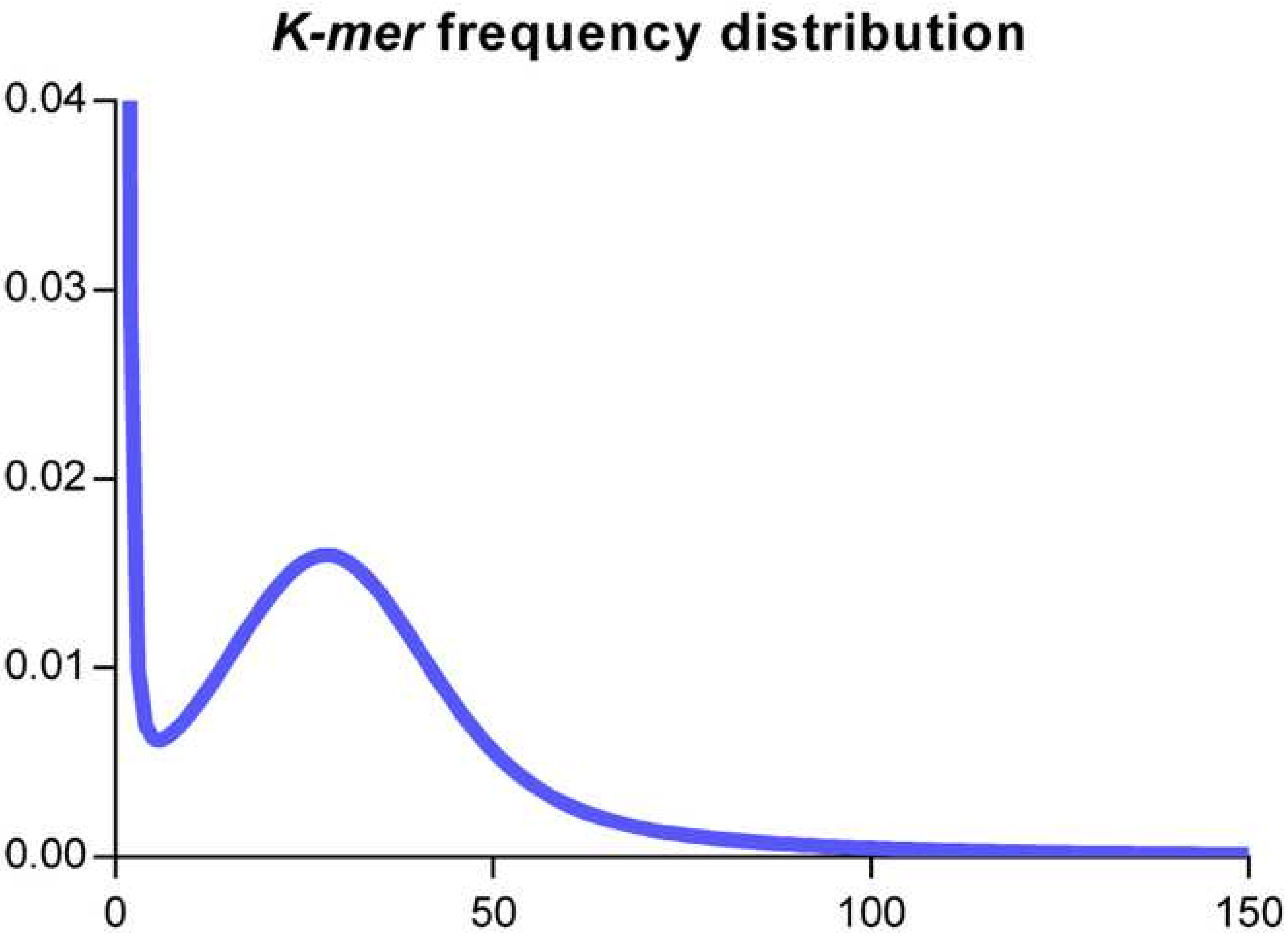

Fig 3

Click here to access/download;Figure;Fig 3.tif

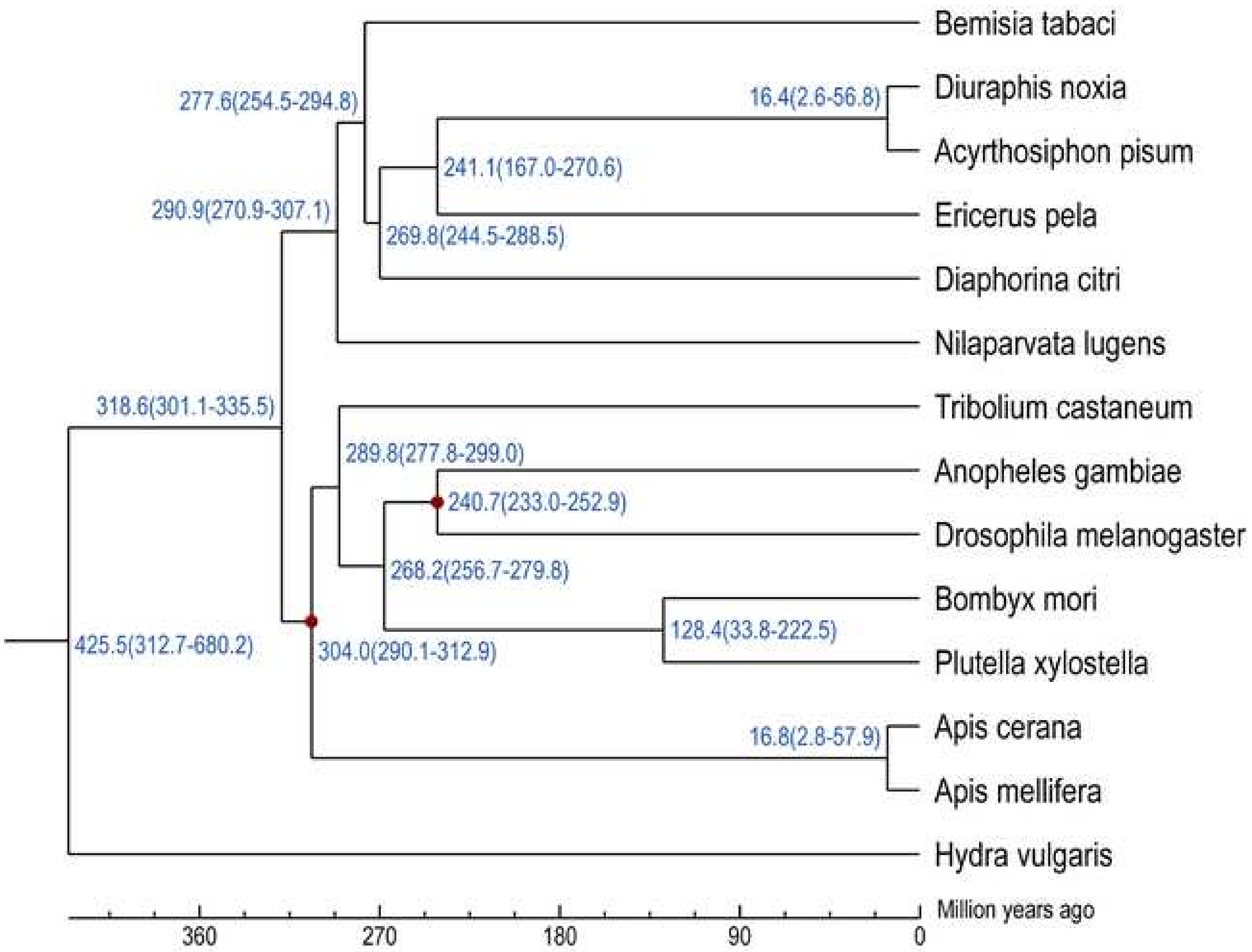

Fig 4

Click here to access/download;Figure;Fig 4.tif

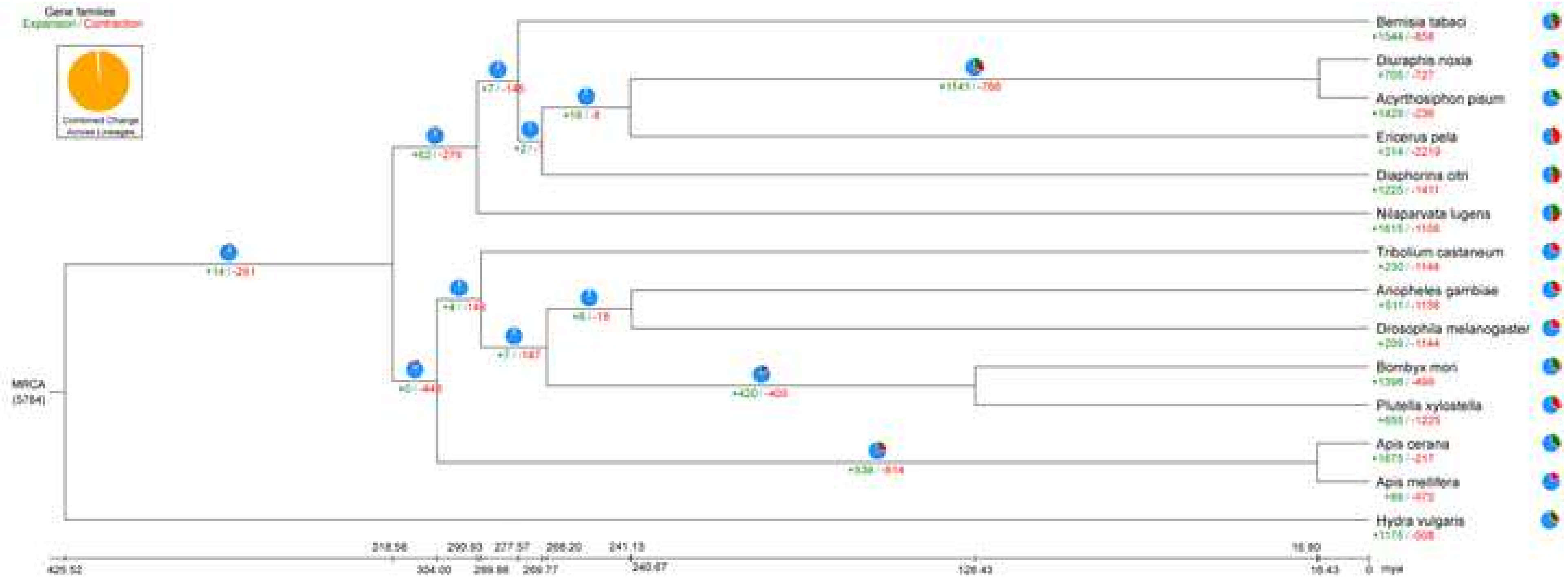

Supplementary Table S1

Click here to access/download
Supplementary Material
Supplementary Table S1.xlsx

Supplementary Table S2

Click here to access/download
Supplementary Material
Supplementary Table S2.xlsx

Supplementary Table S3

Click here to access/download
Supplementary Material
Supplementary Table S3.xlsx

Supplementary Table S4

Click here to access/download
Supplementary Material
Supplementary Table S4.xlsx

Supplementary Table S5

Click here to access/download
Supplementary Material
Supplementary Table S5.xlsx

Supplementary Table S6

Click here to access/download
Supplementary Material
Supplementary Table S6.xlsx

Supplementary Table S7

Click here to access/download
Supplementary Material
Supplementary Table S7.xlsx

Supplementary Fig S1

Click here to access/download
Supplementary Material
Supplementary Fig S1.tif

Supplementary Fig S2

Click here to access/download
Supplementary Material
Supplementary Fig S2.tif

Supplementary Fig S3

Click here to access/download
Supplementary Material
Supplementary Fig S3.tif

Supplementary Fig S4

Click here to access/download
Supplementary Material
Supplementary Fig S4.jpg

Cover Letter Click here to access/download;Personal Cover;Cover Letter.doc

24 SEP. 2018

Editors

GigaScience

Dear editors,

We are submitting a manuscript entitled "Genome Sequence of the Chinese White Wax Scale Insect, the First Draft Genome of the Scale Insects" for your consideration to be published in **GigaScience**.

The Chinese white wax scale insect (*Ericerus pela*) is best known for its important role in producing wax, which has been widely used in candle production, casting, Chinese medicine, and wax printing products over thousands of years. *E. pela* is a typical scale insect. The wax secretion and other unusual features of scale insects are supposed to be adaptation to their ancestral ground-dweller lifestyle and subsequent sedentary lifestyle on high position of plant. In addition to the economic importance, *E. pela* also help understanding the adaptation in scale insects. However, there are no genomic data about *E. pela*. To better understand the genetic information underlying the wax secretion and adaptation of *E. pela*, we assembled the genome of *E. pela*. A total of 303.92 G base pairs (277.22 Gb clean data) were generated using Illumina and Pacbio sequencing. The assembled genome size of *E. pela* was 0.66 Gb with 1,979 scaffold, and the N50 of the scaffold was 735 kb. The *E. pela* genome contained 55.06% repeated sequences. A total of 12,022 protein-coding genes were predicted, with the average CDS length at 1,370 bp. There were 26 fatty acyl-CoA reductase genes and 35 acyltransferase genes which may related to white wax biosynthesis identified. Evolutionary analysis showed that *E. pela* and aphid formed a sister group and split approximately 241.1 million years ago. There were 214 expanded gene families and 2,219 contracted gene families in *E. pela*. Many expanded genes were related to lipid metabolism, and the aldo-keto reductase family expanded significantly in *E. pela* when compared with other insects. The results provide important information and may shed light on the mechanism underlying the wax secretion characteristic of scale insects and the evolution of some unique features of scale insects in exposed living environments.

This work should be of interest to a broad readership. So we submit the present work to your journal.

There are no issues relating to journal policies. We declare that there are no potential competing interests.

We declare that the content of this manuscript has not been published or is not under consideration elsewhere. All authors have agreed to this submission.

Yours sincerely,

Pu Yang
Research Institute of Resource Insects
Chinese Academy of Forestry
Kunming 650224, China
