# Supplementary figures and images for: Genome sequence of the Chinese white wax scale insect Ericerus pela: the first draft genome for the Coccidae family of scale insects
Source: Gigascience. 2019 Sep 13;8(9):giz113. doi: 10.1093/gigascience/giz113 (PMC6743827; doi:10.1093/gigascience/giz113)

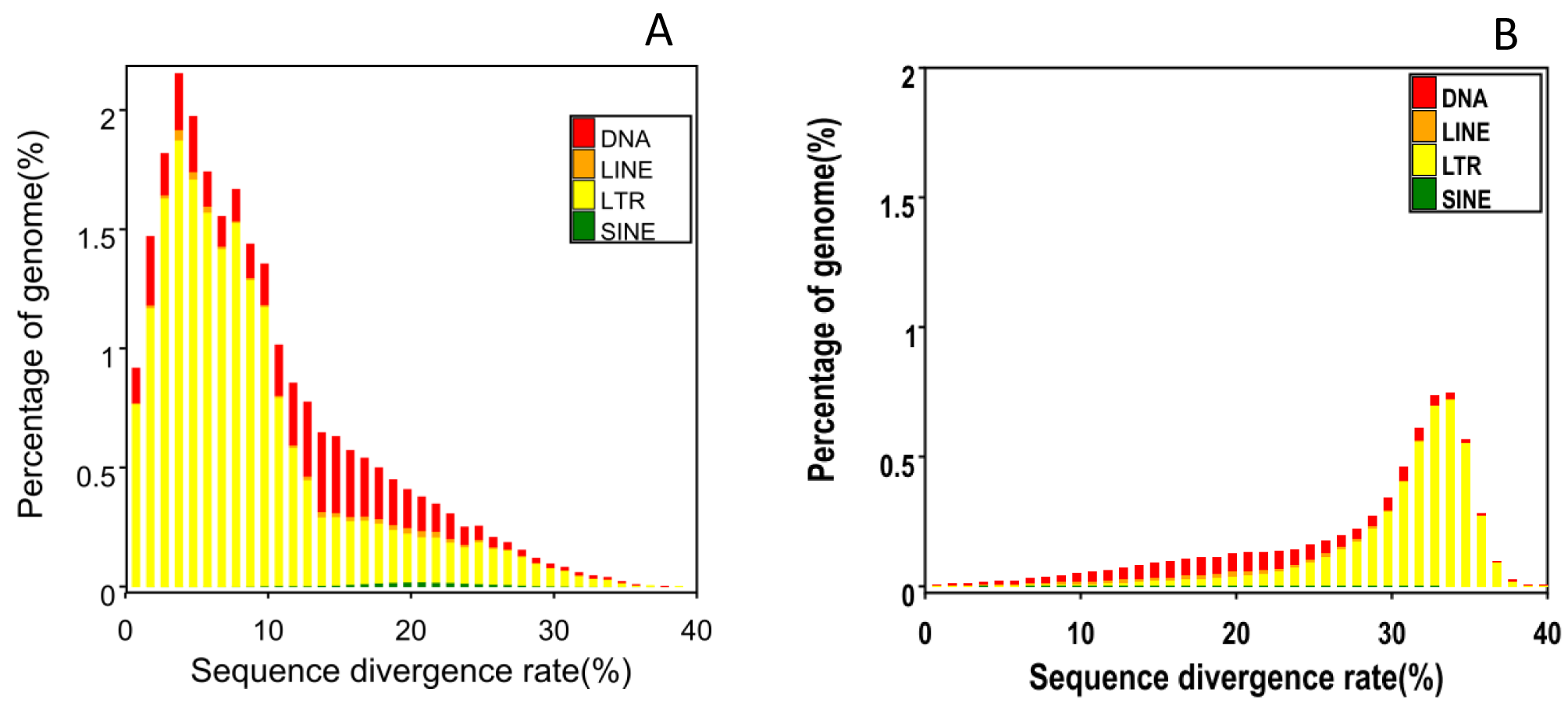

Supplement: giz113_Supplemental_Files [file giz113_supplemental_files.zip › Supplementary Fig S1.tif]

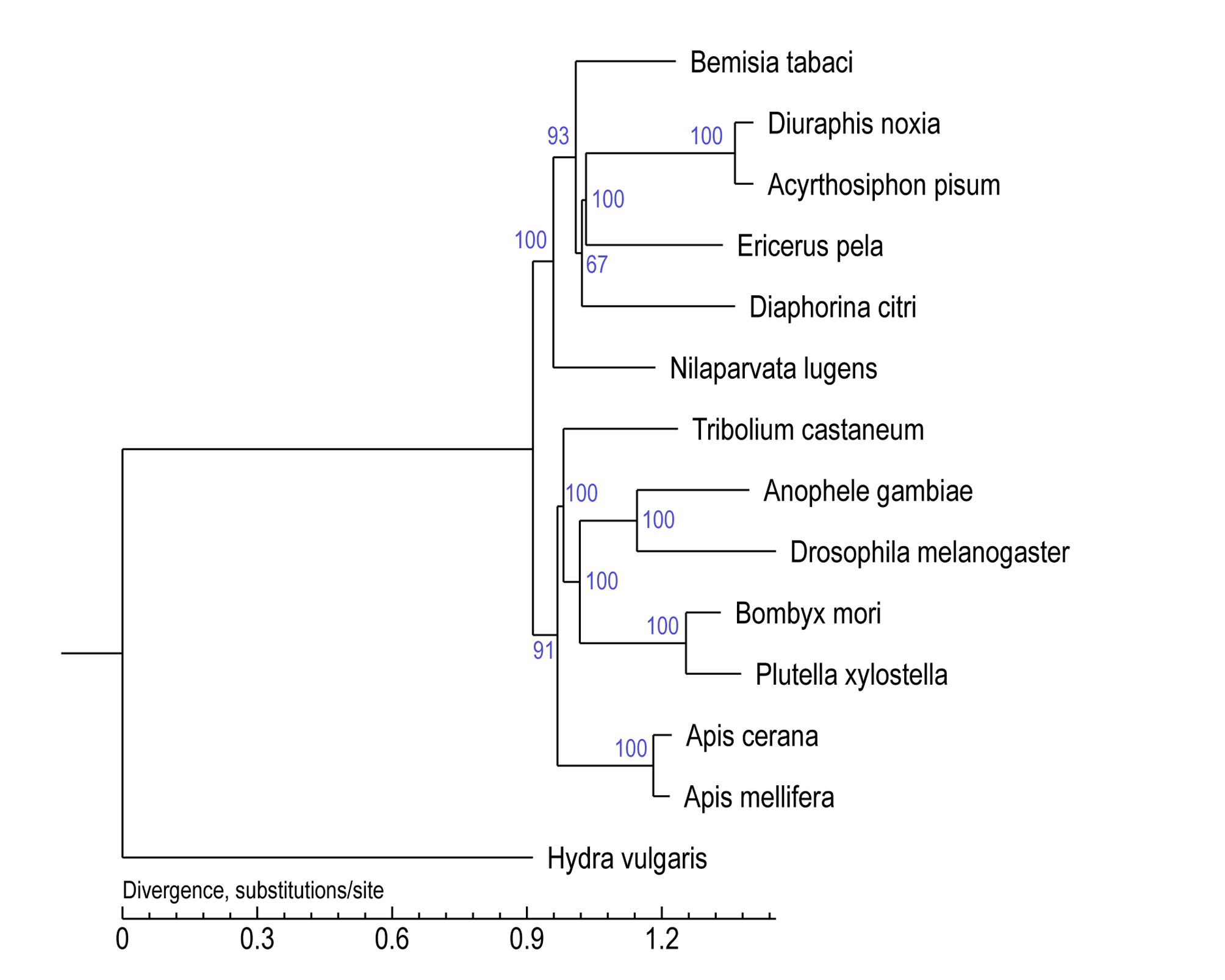

Supplement: giz113_Supplemental_Files [file giz113_supplemental_files.zip › Supplementary Fig S2.tif]

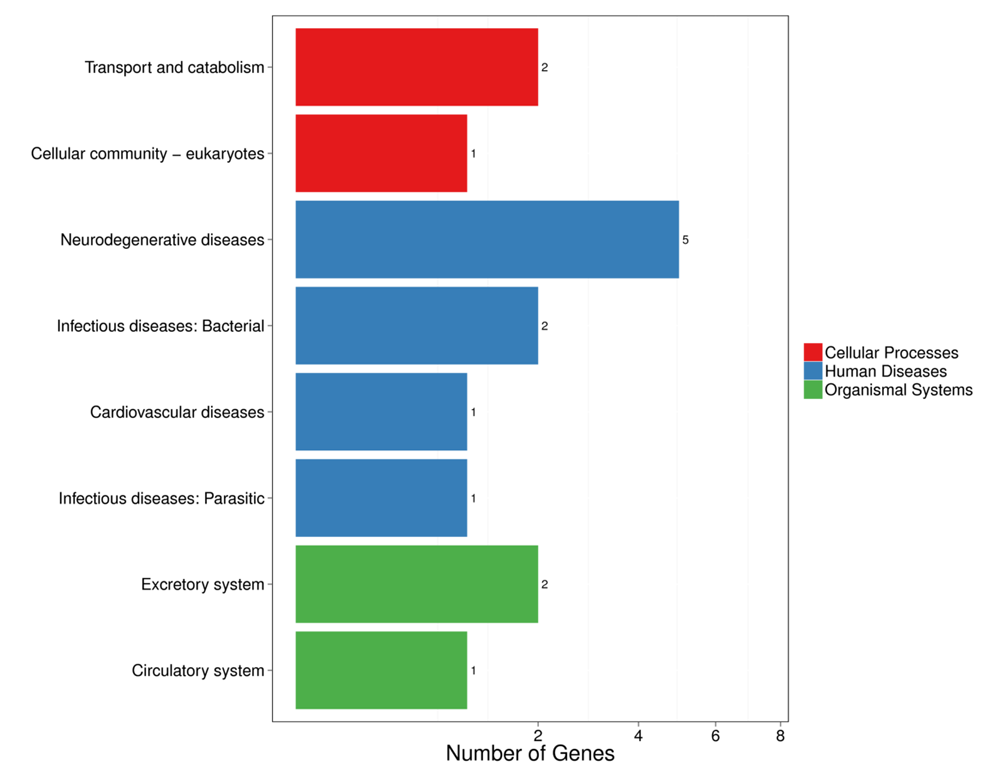

Supplement: giz113_Supplemental_Files [file giz113_supplemental_files.zip › Supplementary Fig S3.tif]

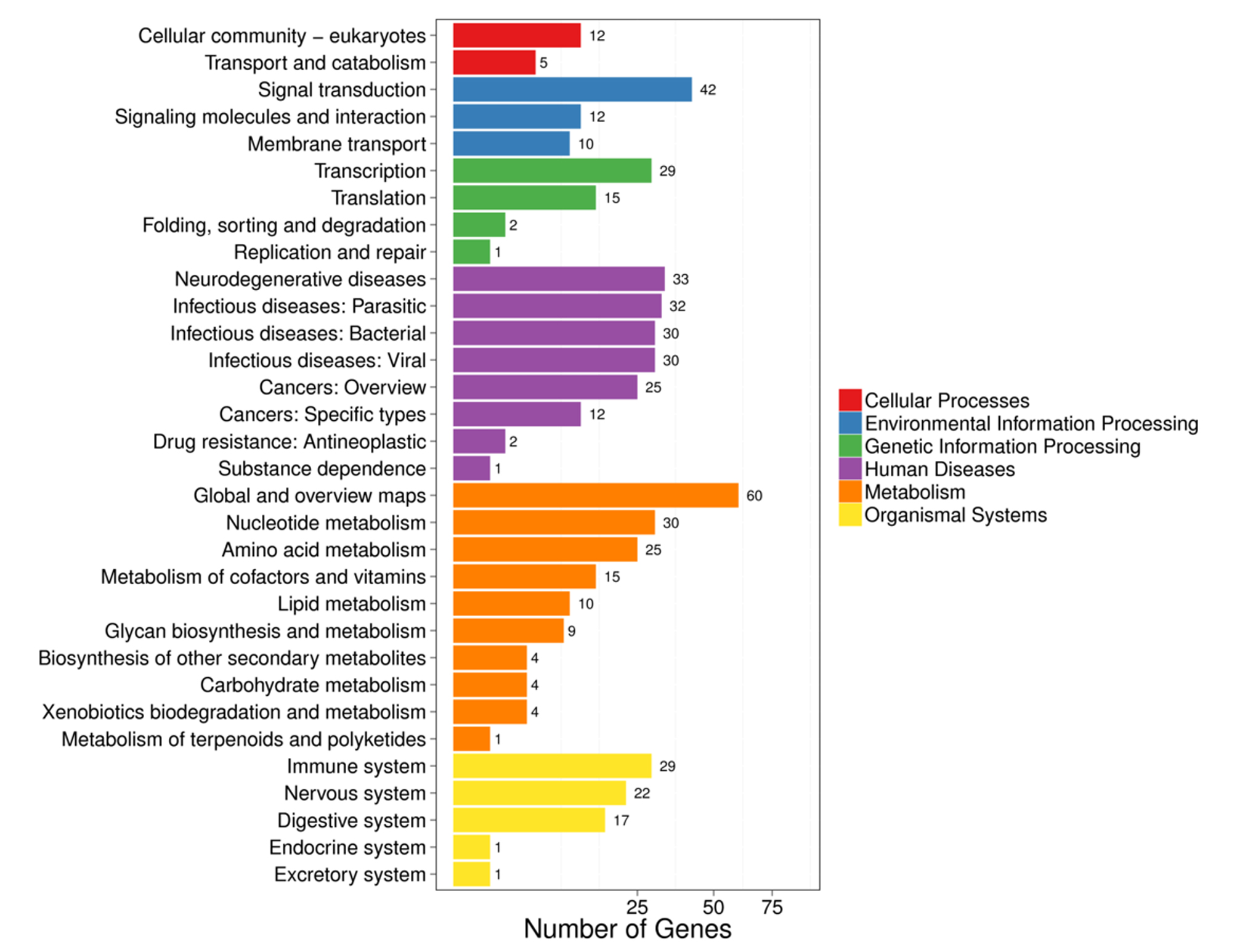

Supplement: giz113_Supplemental_Files [file giz113_supplemental_files.zip › Supplementary Fig S4.jpg]
