# Supplementary material for: Genome sequence of the Chinese white wax scale insect Ericerus pela: the first draft genome for the Coccidae family of scale insects
Source: Gigascience. 2019 Sep 13;8(9):giz113. doi: 10.1093/gigascience/giz113 (PMC6743827; doi:10.1093/gigascience/giz113)
Supplement: giz113_Response_to_Reviewer_Comments_Original_Submission [file giz113_response_to_reviewer_comments_original_submission.pdf]

Author's Response To Reviewer Comments

Close

Dear Editors,
We greatly appreciate your feedback. We have added some detailed information to the revision according to the reviewers' comments. The language of the revision has been improved again by the Springer Nature Author Services. The revision has been uploaded to the submission system, and point-by-point responses to the reviewers are given below. If you have any queries, please do not hesitate to contact me.

Response to Reviewers

Dear Reviewers:

We greatly appreciate your comments on our manuscript. Based on the suggestions, we amended the relevant parts in the manuscript as much as possible. Point-by-point responses to your comments are given below.

Reviewer #1:
The manuscript of Yang et al is very straightforward: it describes the sequencing, assembly and annotation of the genome of the scale insect Ericerus pela.
This is a very good timing since very few genomic resources are available from this group of insect (Coccidae) when they have a key phylogenetic position in the insect tree (mostly for Stenorryncha) and with specific biological adaptation such as in biotechnology (wax production). So this genome is important to be published and the manuscript in its whole deserves publication in GigaScience.

Comment 1: However, I suggest that the authors need to largely improve the form of the manuscript in terms of writing. This concerns mainly the abstract, introduction of parts of the discussion (page 13, pages 15 and 16). I thus strongly invite the authors to revisit those parts.
Response: We are very grateful for your careful reading and thoughtful comments on our manuscript. The manuscript was revised again by the Springer Nature Author Services.

I have some other remarks:
Comment 2: The number of predicted gene is low. This needs to be checked more precisely. The different transcriptomes used for annotation (already published), are not well described. How many conditions (4 from what I understood) and how many reads? If the transcriptomes are too low, this might be one explanation of the small number of predicted genes. From the transcriptomes, didn you already detected a low number of transcripts.
Response: Four transcriptomes were used for annotation. One transcriptome included all the developmental stages of males and females. The other three transcriptomes included a mix of individuals of each of the three developmental stages. The numbers of reads for the four transcriptomes are 88,153,000, 13,333,334, 107,035,314, and 29,014,801, respectively. The numbers of transcripts are 157,501, 41,839, 161,005, and 106,874, respectively. This discrepancy may have resulted from different transcripts of one gene.
Some other insects have gene numbers similar to this insect; for example, Drosophila melanogaster has 13,689 genes, Apis mellifera has 10,660 genes, Pediculus humanus has 10,769 genes, Hycleus cichorii has 13,813 genes, and Hycleus phaleratus has 13,725 genes.

Comment 3: As well, the quality control of the assembly by BUSCO is not well explained. Which set of conserved genes did you used (insects? Arthropds?) and which percentage of these genes did you detect in your assembly?
Response: We have added some relevant details to the revision. Our genome was compared with the genomes in the database of Arthropoda. The complete BUSCO score was 83.2%.

Comment 4: Another explanation could concern the proteins of TE. Sometimes, because they belong to TE, they are eliminated from the annotation. Did you check? This issue of gene number is important since it has a strong consequence on the contracted gene families you describe in the manuscript.
Response: We appreciate your thoughtful comments. We identified tandem repeat sequences using TRF software. The interspersed repeat sequences (transposons) were identified using RepeatMasker and RepeatProteinMask based on the Repbase database. De novo prediction was performed using RepeatMasker software, which was based on the database from RepeatModeler software. Nonredundant results were obtained after all the results predicted from the software above were combined and overlapping results were removed. These methods were also used in some other papers. The identified TEs include LINEs, SINEs, and LTRs, among others.

Comment 5: In the methods, you mention that you have removed the symbionts by dissection. You need to check afterwards in your reads whether you have traces of microorganism sequences (by GC content for instance?).
Response: We uploaded the figure of GC content distribution in the supplementary material, because the picture cannot be shown by pasting here. Please check it in the supplementary material. We are very sorry for the inconvenience.

Comment 6: In the methods as well, you mentioned you extracted 10 micrograms of DNA. Is that sufficient to construct all the Illumina and Pacbio libraries?
Response: Approximately 20 μg genomic DNA was used for the construction of Illumina libraries, and approximately 10 μg genomic DNA was used for PacBio sequencing.

Comment 7: Tables S4 and S5 are -to my opinion - not necessary.
Response: We have deleted Table S4 and Table S5 in the revision.

Comment 8: You mention i5k. Please contact i5k@NAL to post your annotated genome on their databank: this will be better than the whole genome at NCBI (you still have to provide the correct accession number of the genome to allow publication).
Response: Thank you for your comment. We have uploaded the annotated genome to the GigaScience Database, and we will contact i5K to post the annotated genome after publication. The NCBI accession number is QBOQ00000000 and the BioProject number is PRJNA448657 for Ericerus pela.

Comment 9: The legends of the figures are a bit short, and I did not see legends of the supplementary figures.
Response: We have added some information to the figure legends and have added legends for the supplementary figures.

Comment 10: Table 2: change the unit in kb
Response: We appreciate your thoughtful comment. We have changed the unit to kb.

Comment 11: Table 3 could be removed
Response: We have removed Table 3.

Comment 12: Quality of figure 4 is poor
Response: We have improved the quality of Fig. 4.

Thank you for your helpful comments.

Reviewer #2:
The manuscript describes the genome of E. pela, the first Coccidae to have its genome sequenced and assembled, and is generally in well-written English. I don't see any issues with the core principal of the manuscript. I have however some major issues with the methods for gene prediction, and the resulting downstream analysis in the rest of the paper.

Comment 1: I also want to point out that at least all the computational analysis is not explained in enough detail and caused a lot of confusion. If this paper is to be accepted, the entire methods section should be a lot more detailed. E.g. they don't provide enough details on their BLASTN, Augustus, and RNA-Seq based gene prediction, and they only have one sentence on BUSCO gene completeness

assessment (which according to the files provided is actually a genome completeness assessment).
Response: These comments are very helpful for strengthening the paper. We have added some details about gene prediction. We also added some details about the BUSCO gene completeness assessment.

Major points
Comment 2: On page 11 line 36 to 50 the authors state they use BLASTN and Augustus for gene prediction, and use RNA-Seq data to complement this gene prediction. However, the authors don't go into detail, and are very vague. From what I gather, they use BLASTN, RNA-Seq, and Augustus separately from each other instead of using BLASTN and RNA-Seq results as a hints file for Augustus*.
Response: We apologize for the lack of details. We used blast with the genomes of eight species as references for homology prediction. The complete gene sets were selected separately from the homology prediction results, and 2,000 gene sets were selected at random from the eight species; complete genes were selected for Augustus training. The RNA-seq data were used to supplement the gene sets. We have added this information to the revision.

Comment 3: I have several issues with this:
1) Using BLASTN by itself to predict genes is prone to finding only partial genes, and only finding genes for which there is a homolog publicly available.
Response: We agree. In addition to BLASTN, we used Augustus software for de novo prediction and used RNA-seq data for gene prediction. The predicted genes were integrated into one non-redundant and more complete gene set.

Comment 4: 2) Augustus uses pre-trained models to predict genes. The authors used an Aphid species (Acyrthosiphon pisum) as the pre-trained model as this is the closest one available to E. pela. However, aphids are phylogeneticly too distant of a species to use as a reliable input for E. pela gene prediction with Augustus. I suspect that Augustus therefore only predicts a limited amount of genes for E. pela. Table 3 also shows a very high divergence between Augustus results when using different models. As a side-note, the authors state themselves aphids and scale insects likely diverged more than 200 million years ago, which proves the point that both species are likely too distant to use as a reference for gene prediction.
Response: We apologize for the lack of detailed information in the methods section. We used Augustus for gene prediction as follows: the genomes of eight species were selected as references for homology prediction. Then, the complete gene sets were selected separately from the homology prediction results. In addition, 2,000 gene sets were selected at random from the eight species, and complete genes were selected for Augustus training. The Augustus prediction result for the E. pela genome was 19,941 genes. We have added these details to the revision.

Comment 5: 3) RNA-Seq, like BLASTN, is likely to give many partial genes. Also, many genes might be missed due to low or lack of expression.
Response: We agree. A transcriptome including all the developmental stages was used, and the three other transcriptomes included each of the three developmental stages. Furthermore, in addition to RNA-seq, we used Augustus software and BLASTN for gene prediction.

Comment 6: Because of these three points I think it might be that the authors are missing a large amount of genes.
Response: The methods we used for gene prediction were also used in other studies, such as the following:
Shifeng Cheng et al., De novo assembly of the Mongolian gerbil genome and transcriptome. bioRxiv 2019;
Wang et al. Genome sequence of a rice pest, the white-backed planthopper (Sogatella furcifera). GigaScience 2017, 6: 1-9;
Chen et al. High-quality genome assembly of channel catfish, Ictalurus punctatus. GigaScience 2016, 5:39;
Xue et al. Genomes of the rice pest brown planthopper and its endosymbionts reveal complex complementary contributions for host adaptation. Genome Biology 2014, 15:521;
Kocher et al. The draft genome of a socially polymorphic halictid bee, Lasioglossum albipes. Genome Biology 2013, 14: R142.
Some hemimetabolous and holometabolous insects also have slightly more than ten thousand genes; for example, Drosophila melanogaster has 13,689 genes, Apis mellifera has 10,660 genes, and Pediculus humanus has 10,769 genes, Hycleus cichorii has 13,813 genes, and Hycleus phaleratus has 13,725 genes.

Comment 7: This could also explain the low amount of genes compared to other hemiptera (page 12 line 4-15), and the high amount of genes/proteins with a 'functional annotation'** (page 12 line 12-15). The amount of proteins that falls under a cateory given by table 4 is unrealistically high, considering E. pela is the first Coccidae genome. Usually proteins from a species for which the phylogenetic close-by species has the only, or one of the few, sequenced genomes finds orthologous proteins with a lower rate than usual (anecdotal from my side, more around 50%). So to find such a high rate of features annotated to the E. pela proteins is surprising.
It might be that E. pela is lucky and does indeed find most of its proteins to have a documented ortholog, but also considering the previous point on gene prediction, I think the authors are currently dealing with a smaller, more conserved part of the E. pela proteome.
Response: Thank you for your comment. We think with more and more species were genome sequenced, the percent of genes with functional annotation is high. Some insects has high amount of genes with functional annotation. For example, 88.44% of genes were annotated with one or more related functions in Pyrocoelia pectoralis, 80.68% of genes were assigned with at least one related function in Stenopsyche tienmushanensis, 88.82% of genes were annotated with one or more related functions in Hycleus cichorii, 89.22% of genes were annotated with one or more related functions in H. phaleratus.
Luo et al., 2018. The genome of an underwater architect, the caddisfly Stenopsyche tienmushanensis Hwang (Insecta: Trichoptera). GigaScience, 7: 1–12.
Wu et al., 2018. Draft genomes of two blister beetles Hycleus cichorii and Hycleus phaleratus. GigaScience, 7: 1–7.
Fu et al., 2017. Long-read sequence assembly of the firefly Pyrocoelia pectoralis genome. GigaScience, 0: 1–7.

Comment 8: As a check, I did a quick GeneMark-ET (self-trained) on the genome file. It gave 47,125 potential genes. This is likely a gross overestimation, but inputting these into an Augustus hints file followed by Augustus gene prediction will likely filter out most false positives and give you a much more realistic number of genes.
I would like the authors to redo their gene prediction using non-homology methods, e.g. using GeneMark ET self-training, and by using their BLASTN and RNA-Seq results as a hints file input to Augustus. The GeneMark ET self-training results might also serve as hints input to Augustus. If the gene predictions do not significantly differ with their current gene predictions, the authors are indeed dealing with a more-or-less correct gene-set / proteome of E. pela. However, if it differs significantly, they will have to take an in-depth look at using the new genes, or merging them with the previous genes.
Response: We appreciate your thoughtful comments on our manuscript. We used the GeneMark-ET self-training method, and the predicted gene number was 91,350. GeneMark-ES predicted 46,784 genes. The details are shown in a table as below:
Type(#) Total Number(#) Total Length (bp) Average Length (#) Length/Genome Length (%)
Gene Stat 46,784 275,703,996 5,893.13 41.72
Exons Stat 257,481 94,734,433 367.93 14.33
CDS Stat 46,784 94,734,433 2,024.93 14.33
Introne Stat 210,697 180,969,563 858.91 27.3

We merged the GeneMark-ES result (46,784) with Augustus result. The final result of genes number is 45,984. The details are shown in a table as below:
Type(#) Total Number (#) Total Length (bp) Average Length (#) Length/Genome Length (%)
Gene Stat 45,984 273,191,552 5,941.01 41.37
Exons Stat 253,134 93,262,275 368.43 14.12
CDS Stat 45,984 93,262,275 2,028.15 14.12
Introne Stat 207,150 179,929,277 868.59 27.24

The gene number (45,984) is larger than those for many insects. In addition, the percent of CDS length to genome length is 14%, which is higher than that of animals. We appreciate your comment. Perhaps we need to use many methods to predict genes, which may require many attempts. We previously used the BLAST, Augustus, and RNA-seq methods, which have also been used in other studies.

Comment 9: This would also require the authors to redo their whole downstream analysis, as this is all based on the assumption that the gene set they have is accurate. I do not believe redoing this analysis, including the gene prediction, is extremely time consuming and think the authors should be able to do this in a reasonable time frame.
Response: Thank you for your thoughtful comment. We have tried to use the method of GeneMark and Augustus. The predicted gene number is 45,984. The number of genes is 12,022 in our previous

prediction. Many insects have slightly more than ten thousand genes. The methods we used for gene prediction were also used in other studies. We will try to update the genome in the future if more scientific methods for sequencing and annotating the genome are available.

Comment 10: * Table 3 shows the authors also predicted genes with MAKER, but no mention has been made of this in the main text. The authors should clarify why they used MAKER, and if they used the MAKER output in their final results.
Response: We apologize for the lack of details. MAKER software was used for self-training. The returned gene number was low and was not used in the final gene sets. Thus, we have deleted it.

Comment 11: ** Table 4 shows the functional annotation of E. pela. However, I think the authors miss-use the definition of 'functional annotation' here. All categories except for 'GO-Annotated' don't necessarily provide a functional annotation, as functional annotations are usually GO terms. The authors state that 87.99% of the proteins have a functional annotation, but they do this by counting every protein that falls under one of the categories in table 4. The category of GO-annotated proteins is only 32.23%.
Response: We counted every protein that fell into one of the categories (Nr, SwissProt, KEGG, and GO, among others). KEGG annotation is an important method of functional annotation, and 71.77% of the proteins were functionally annotated.

Comment 12: Page 10 line 45-46. The authors state they used BUSCO to asses coding gene completeness but they don't mention a number or any other details. The BUSCO summary file is available but should be mentioned in the text.
Response: We have added some details about the BUSCO results to the revision.

Comment 13: Additionally the authors state they assessed coding gene completeness, but the command line in '9_BUSCO_output_full_table_arthropoda" has '-m genome' as an option, which means they assessed genome completeness not gene completeness.
Response: Thank you for your careful read. Our genome was compared with the database of Arthropoda genomes. The complete BUSCO score was 83.2%.

Comment 14: Finally, the authors BUSCO Augustus gene prediction uses fly (drosophila) instead of the aphid they used in their own Augustus gene prediction. If they want to do a genome completeness assessment with Busco the authors should change it to the aphid.
Response: We have added detailed information to the methods section in the revision. We used Augustus software for de novo gene prediction. We performed a genome completeness assessment with the Arthropoda database by using BUSCO.

Minor points
Comment 15: Page 13, Line 17 to 20
Did the authors trim poorly aligned sequence regions in the after multiple-sequence alignment and before the creation of 'super genes'? If not, it is likely that these poorly aligned regions influence the phylogenetic tree in a negative way.
Response: Yes, the sequences were filtered by percent match length with a cut-off of 50%.

Comment 16: Figure 3. I think it would be best to show the bootstrap values. Right now they are buried in the supplementary.
Response: The tree of divergence times and the tree of gene family contraction and expansion were constructed based on the phylogenetic results. The bootstrap values are shown in the phylogenetic tree. Because there were divergence times or gene family numbers in the other two trees, the bootstraps were not shown.

Comment 17: Figure 4. Cafe gives a p-value to see if there is a significant change in expansion or contraction. The authors should provide these.
Response: We used a p-value of 0.05. We have added this information to the revision.

Comment 18: GO Terms and other annotations to the genes/proteins. I can't find them. Where are they?
Response: We will upload the annotation results through FTP.

Thank you for your helpful comments.

Close
