# Supplementary material for: Genome sequence of the Chinese white wax scale insect Ericerus pela: the first draft genome for the Coccidae family of scale insects
Source: Gigascience. 2019 Sep 13;8(9):giz113. doi: 10.1093/gigascience/giz113 (PMC6743827; doi:10.1093/gigascience/giz113)
Supplement: giz113_Response_to_Reviewer_Comments_Revision_1 [file giz113_response_to_reviewer_comments_revision_1.pdf]

Author's Response To Reviewer Comments

Close

Dear Editors,
Thank you so much for your kind work for us. We have added some detailed information to the revision according to the reviewers’ comments. The revision has been uploaded to the submission system. The point-by-point responses to the reviewers are given below. There is no new software application used in the manuscript. Should you have any inquiries, please contact me without any hesitation.

Response to Reviewers

Dear Reviewers:

Thank you so much for your comments on our manuscript. Based on your suggestions, we amended the relevant parts in the manuscript as much as possible. Point-by-point responses to your comments are given below.

Reviewer #2:
Dear authors,
Sorry for the delay on the review. Thank you for putting in the work for clarifying your methodologies, which answers most of my comments.
I just have two minor comments on your response:

Comment 1: When I proposed doing the GeneMark-ET (or ES) I envisioned, as explained, to use these genes as an input for Augustus to further curate these genes. I agree that by simply merging the results with what you already had, you get an overestimation of the actual amount of genes. However, given the rest of your clarifications, I don't see this original comment of mine now as necessary.
Response: We greatly appreciate your thoughtful comments on our manuscript. We have tried to do that, and we agree.

Comment 2: You responded several times with 'They did the same in paper X and X and X' when it comes to gene prediction, but this does not mean that this (and their) methodology is correct. I am still not _entirely_ convinced this is a correct way of doing it, but given the rest of the comments, I believe everything is in order.
Response: We are sorry for the response with that. We have tried to use GeneMark and merge the results. We will try to update the genome in the future if there are more scientific methods are available.

Comment 3: Also, I am not sure if you mention which 8 species you use as homologues for gene prediction, although I might have missed it. If it is indeed missing, please add it to the text.
Response: Thank you for your thoughtful comments on our manuscript. The eight species we used were Acyrthosiphon pisum, Apis mellifera, Bemisia tabaci, Bombyx mori, Drosophila melanogaster, Nasonia vitripennis, Pediculus humanus, Tribolium castaneum. We have added it to the revision.

Once again, thank you very much for your helpful comments.

Close
