# Supplementary material for: Genome sequence of the Chinese white wax scale insect Ericerus pela: the first draft genome for the Coccidae family of scale insects
Source: Gigascience. 2019 Sep 13;8(9):giz113. doi: 10.1093/gigascience/giz113 (PMC6743827; doi:10.1093/gigascience/giz113)
Supplement: giz113_Response_to_Reviewer_Comments_Revision_2 [file giz113_response_to_reviewer_comments_revision_2.pdf]

Author's Response To Reviewer Comments

Close

Dear Editors,
Thank you so much for your kind work for us. We greatly appreciate your thoughtful comments and the edits you have done for us. We have checked all the edits and switched the track changes off. The manuscript has been resubmitted through the system.

Close
