# Supplementary material for: Genome sequence of the Chinese white wax scale insect Ericerus pela: the first draft genome for the Coccidae family of scale insects
Source: Gigascience. 2019 Sep 13;8(9):giz113. doi: 10.1093/gigascience/giz113 (PMC6743827; doi:10.1093/gigascience/giz113)
Supplement: giz113_Reviewer_1_Report_Original_Submission — Denis Tagu -- 11/30/2018 Reviewed [file giz113_reviewer_1_report_original_submission.pdf]

Reviewer Report

Title: Genome Sequence of the Chinese White Wax Scale Insect, Ericerus pela: the First Draft Genome for the Coccidae Family of Scale Insects

Version: Original Submission **Date:** 11/30/2018

Reviewer name: Denis Tagu

Reviewer Comments to Author:

The manuscript of Yang et al is very straightforward: it describes the sequencing, assembly and annotation of the genome of the scale insect Ericerus pela.
This is a very good timing since very few genomic resources are available from this group of insect (Coccidae) when they have a key phylogenetic position in the insect tree (mostly for Stenorryncha) and with specific biological adaptation such as in biotechnology (wax production). So this genome is important to be published and the manuscript in its whole deserves publication in GigaScience.
However, I suggest that the authors need to largely improve the form of the manuscript in terms of writing. This concerns mainly the abstract, introduction of parts of the discussion (page 13, pages 15 and 16). I thus strongly invite the authors to revisit those parts.
I have some other remarks:
The number of predicted gene is low. This needs to be checked more precisely. The different transcriptomes used for annotation (already published), are not well described. How many conditions (4 from what I understood) and how many reads? If the transcriptomes are too low, this might be one explanation of the small number of predicted genes. From the transcriptomes, didn you already detected a low number of transcripts. As well, the quality control of the assembly by BUSCO is not well explained. Which set of conserved genes did you used (insects? Arthropds?) and which percentage of these genes did you detect in your assembly? Another explanation could concern the proteins of TE. Sometimes, because they belong to TE, they are eliminated from the annotation. Did you check? This issue of gene number is important since it has a strong consequence on the contracted gene families you describe in the manuscript.
In the methods, you mention that you have removed the symbionts by dissection. You need to check afterwards in your reads whether you have traces of microorganism sequences (by GC content for instance?).
In the methods as well, you mentioned you extracted 10 micrograms of DNA. Is that sufficient to construct all the Illumina and Pacbio libraries?
Tables S4 and S5 are -to my opinion - not necessary.
You mention i5k. Please contact i5k@NAL to post your annotated genome on their databank: this will be better than the whole genome at NCBI (you still have to provide the correct accession number of the genome to allow publication).
The legends of the figures are a bit short, and I did not see legends of the supplementary figures.
Table 2: change the unit in kb
Table 3 could be removed

Quality of figure 4 is poor

Level of Interest

Please indicate how interesting you found the manuscript: Choose an item.

Quality of Written English

Please indicate the quality of language in the manuscript: Choose an item.

Declaration of Competing Interests

Please complete a declaration of competing interests, considering the following questions:

- Have you in the past five years received reimbursements, fees, funding, or salary from an organisation that may in any way gain or lose financially from the publication of this manuscript, either now or in the future?
- Do you hold any stocks or shares in an organisation that may in any way gain or lose financially from the publication of this manuscript, either now or in the future?
- Do you hold or are you currently applying for any patents relating to the content of the manuscript?
- Have you received reimbursements, fees, funding, or salary from an organization that holds or has applied for patents relating to the content of the manuscript?
- Do you have any other financial competing interests?
- Do you have any non-financial competing interests in relation to this paper?

If you can answer no to all of the above, write 'I declare that I have no competing interests' below. If your reply is yes to any, please give details below.

I declare that I have no competing interests

I agree to the open peer review policy of the journal. I understand that my name will be included on my report to the authors and, if the manuscript is accepted for publication, my named report including any attachments I upload will be posted on the website along with the authors' responses. I agree for my report to be made available under an Open Access Creative Commons CC-BY license (http://creativecommons.org/licenses/by/4.0/). I understand that any comments which I do not wish to be included in my named report can be included as confidential comments to the editors, which will not be published.

Choose an item.

To further support our reviewers, we have joined with Publons, where you can gain additional credit to further highlight your hard work (see: https://publons.com/journal/530/gigascience). On publication of this paper, your review will be automatically added to Publons, you can then choose whether or not to claim your Publons credit. I understand this statement.

Yes Choose an item.
