# Supplementary material for: Genome sequence of the Chinese white wax scale insect Ericerus pela: the first draft genome for the Coccidae family of scale insects
Source: Gigascience. 2019 Sep 13;8(9):giz113. doi: 10.1093/gigascience/giz113 (PMC6743827; doi:10.1093/gigascience/giz113)
Supplement: giz113_Reviewer_2_Report_Original_Submission — Maarten Reijnders -- 4/5/2019 Reviewed [file giz113_reviewer_2_report_original_submission.pdf]

Reviewer Report

Title: Genome Sequence of the Chinese White Wax Scale Insect, Ericerus pela: the First Draft Genome for the Coccidae Family of Scale Insects

Version: Original Submission **Date:** 4/5/2019

Reviewer name: Maarten Reijnders

Reviewer Comments to Author:

The manuscript describes the genome of E. pela, the first Coccidae to have its genome sequenced and assembled, and is generally in well-written English. I don't see any issues with the core principal of the manuscript. I have however some major issues with the methods for gene prediction, and the resulting downstream analysis in the rest of the paper.
I also want to point out that at least all the computational analysis is not explained in enough detail and caused a lot of confusion. If this paper is to be accepted, the entire methods section should be a lot more detailed. E.g. they don't provide enough details on their BLASTN, Augustus, and RNA-Seq based gene prediction, and they only have one sentence on BUSCO gene completeness assessment (which according to the files provided is actually a genome completeness assessment).
Major points
On page 11 line 36 to 50 the authors state they use BLASTN and Augustus for gene prediction, and use RNA-Seq data to complement this gene prediction. However, the authors don't go into detail, and are very vague. From what I gather, they use BLASTN, RNA-Seq, and Augustus separately from each other instead of using BLASTN and RNA-Seq results as a hints file for Augustus*. I have several issues with this:
1) Using BLASTN by itself to predict genes is prone to finding only partial genes, and only finding genes for which there is a homolog publicly available.
2) Augustus uses pre-trained models to predict genes. The authors used an Aphid species (Acyrthosiphon pisum) as the pre-trained model as this is the closest one available to E. pela. However, aphids are phylogeneticly too distant of a species to use as a reliable input for E. pela gene prediction with Augustus. I suspect that Augustus therefore only predicts a limited amount of genes for E. pela. Table 3 also shows a very high divergence between Augustus results when using different models. As a side-note, the authors state themselves aphids and scale insects likely diverged more than 200 million years ago, which proves the point that both species are likely too distant to use as a reference for gene prediction.
3) RNA-Seq, like BLASTN, is likely to give many partial genes. Also, many genes might be missed due to low or lack of expression.
Because of these three points I think it might be that the authors are missing a large amount of genes. This could also explain the low amount of genes compared to other hemiptera (page 12 line 4-15), and the high amount of genes/proteins with a 'functional annotation'** (page 12 line 12-15). The amount of proteins that falls under a cateory given by table 4 is unrealistically high, considering E. pela is the first Coccidae genome. Usually proteins from a species for which the phylogenetic close-by species has the only, or one of the few, sequenced genomes finds orthologous proteins with a lower rate than usual

(anecdotal from my side, more around 50%). So to find such a high rate of features annotated to the E. pela proteins is surprising.
It might be that E. pela is lucky and does indeed find most of its proteins to have a documented ortholog, but also considering the previous point on gene prediction, I think the authors are currently dealing with a smaller, more conserved part of the E. pela proteome.
As a check, I did a quick GeneMark-ET (self-trained) on the genome file. It gave 47,125 potential genes. This is likely a gross overestimation, but inputting these into an Augustus hints file followed by Augustus gene prediction will likely filter out most false positives and give you a much more realistic number of genes.
I would like the authors to redo their gene prediction using non-homology methods, e.g. using GeneMark ET self-training, and by using their BLASTN and RNA-Seq results as a hints file input to Augustus. The GeneMark ET self-training results might also serve as hints input to Augustus. If the gene predictions do not significantly differ with their current gene predictions, the authors are indeed dealing with a more-or-less correct gene-set / proteome of E. pela. However, if it differs significantly, they will have to take an in-depth look at using the new genes, or merging them with the previous genes.
This would also require the authors to redo their whole downstream analysis, as this is all based on the assumption that the gene set they have is accurate. I do not believe redoing this analysis, including the gene prediction, is extremely time consuming and think the authors should be able to do this in a reasonable time frame.
* Table 3 shows the authors also predicted genes with MAKER, but no mention has been made of this in the main text. The authors should clarify why they used MAKER, and if they used the MAKER output in their final results.
** Table 4 shows the functional annotation of E. pela. However, I think the authors miss-use the definition of 'functional annotation' here. All categories except for 'GO-Annotated' don't necessarily provide a functional annotation, as functional annotations are usually GO terms. The authors state that 87.99% of the proteins have a functional annotation, but they do this by counting every protein that falls under one of the categories in table 4. The category of GO-annotated proteins is only 32.23%.
Page 10 line 45-46
The authors state they used BUSCO to asses coding gene completeness but they don't mention a number or any other details. The BUSCO summary file is available but should be mentioned in the text. Additionally the authors state they assessed coding gene completeness, but the command line in '9_BUSCO_output_full_table_arthropoda" has '-m genome' as an option, which means they assessed genome completeness not gene completeness.
Finally, the authors BUSCO Augustus gene prediction uses fly (drosophila) instead of the aphid they used in their own Augustus gene prediction. If they want to do a genome completeness assessment with Busco the authors should change it to the aphid.
Minor points
Page 13
Line 17 to 20
Did the authors trim poorly aligned sequence regions in the after multiple-sequence alignment and before the creation of 'super genes'? If not, it is likely that these poorly aligned regions influence the phylogenetic tree in a negative way.

Figure 3
I think it would be best to show the bootstrap values. Right now they are buried in the supplementary.
Figure 4
Cafe gives a p-value to see if there is a significant change in expansion or contraction. The authors should provide these.
GO Terms and other annotations to the genes/proteins
I can't find them. Where are they?

Level of Interest

Please indicate how interesting you found the manuscript: Choose an item.

Quality of Written English

Please indicate the quality of language in the manuscript: Choose an item.

Declaration of Competing Interests

Please complete a declaration of competing interests, considering the following questions:

- Have you in the past five years received reimbursements, fees, funding, or salary from an organisation that may in any way gain or lose financially from the publication of this manuscript, either now or in the future?
- Do you hold any stocks or shares in an organisation that may in any way gain or lose financially from the publication of this manuscript, either now or in the future?
- Do you hold or are you currently applying for any patents relating to the content of the manuscript?
- Have you received reimbursements, fees, funding, or salary from an organization that holds or has applied for patents relating to the content of the manuscript?
- Do you have any other financial competing interests?
- Do you have any non-financial competing interests in relation to this paper?

If you can answer no to all of the above, write 'I declare that I have no competing interests' below. If your reply is yes to any, please give details below.

I declare that I have no competing interests

I agree to the open peer review policy of the journal. I understand that my name will be included on my report to the authors and, if the manuscript is accepted for publication, my named report including any attachments I upload will be posted on the website along with the authors' responses. I agree for my report to be made available under an Open Access Creative Commons CC-BY license

(http://creativecommons.org/licenses/by/4.0/). I understand that any comments which I do not wish to be included in my named report can be included as confidential comments to the editors, which will not be published.

Choose an item.

To further support our reviewers, we have joined with Publons, where you can gain additional credit to further highlight your hard work (see: https://publons.com/journal/530/gigascience). On publication of this paper, your review will be automatically added to Publons, you can then choose whether or not to claim your Publons credit. I understand this statement.

Yes Choose an item.
