# Supplementary material for: Genome sequence of the Chinese white wax scale insect Ericerus pela: the first draft genome for the Coccidae family of scale insects
Source: Gigascience. 2019 Sep 13;8(9):giz113. doi: 10.1093/gigascience/giz113 (PMC6743827; doi:10.1093/gigascience/giz113)
Supplement: giz113_Reviewer_2_Report_Revision_1 — Maarten Reijnders -- 7/4/2019 Reviewed [file giz113_reviewer_2_report_revision_1.pdf]

Reviewer Report

Title: Genome Sequence of the Chinese White Wax Scale Insect, Ericerus pela: the First Draft Genome for the Coccidae Family of Scale Insects

Version: Revision 1 **Date:** 7/4/2019

Reviewer name: Maarten Reijnders

Reviewer Comments to Author:

Dear authors,
Sorry for the delay on the review. Thank you for putting in the work for clarifying your methodologies, which answers most of my comments.
I just have two minor comments on your response:
1) When i proposed doing the GeneMark-ET (or ES) i envisioned, as explained, to use these genes as an input for Augustus to further curate these genes. I agree that by simply merging the results with what you already had, you get an overestimation of the actual amount of genes. However, given the rest of your clarifications, i don't see this original comment of mine now as necessary.
2) You responded several times with 'They did the same in paper X and X and X' when it comes to gene prediction, but this does not mean that this (and their) methodology is correct. I am still not _entirely_ convinced this is a correct way of doing it, but given the rest of the comments, i believe everything is in order.
Also, i am not sure if you mention which 8 species you use as homologues for gene prediction, although i might have missed it. If it is indeed missing, please add it to the text.
Kind regards

Level of Interest

Please indicate how interesting you found the manuscript: Choose an item.

Quality of Written English

Please indicate the quality of language in the manuscript: Choose an item.

Declaration of Competing Interests

Please complete a declaration of competing interests, considering the following questions:

- Have you in the past five years received reimbursements, fees, funding, or salary from an organisation that may in any way gain or lose financially from the publication of this manuscript, either now or in the future?
- Do you hold any stocks or shares in an organisation that may in any way gain or lose financially from the publication of this manuscript, either now or in the future?

- Do you hold or are you currently applying for any patents relating to the content of the manuscript?
- Have you received reimbursements, fees, funding, or salary from an organization that holds or has applied for patents relating to the content of the manuscript?
- Do you have any other financial competing interests?
- Do you have any non-financial competing interests in relation to this paper?

If you can answer no to all of the above, write 'I declare that I have no competing interests' below. If your reply is yes to any, please give details below.

I declare that i have no competing interests

I agree to the open peer review policy of the journal. I understand that my name will be included on my report to the authors and, if the manuscript is accepted for publication, my named report including any attachments I upload will be posted on the website along with the authors' responses. I agree for my report to be made available under an Open Access Creative Commons CC-BY license (http://creativecommons.org/licenses/by/4.0/). I understand that any comments which I do not wish to be included in my named report can be included as confidential comments to the editors, which will not be published.

Choose an item.

To further support our reviewers, we have joined with Publons, where you can gain additional credit to further highlight your hard work (see: https://publons.com/journal/530/gigascience). On publication of this paper, your review will be automatically added to Publons, you can then choose whether or not to claim your Publons credit. I understand this statement.

Yes Choose an item.
